# Diverse Mechanisms of Protective Anti-Pneumococcal Antibodies

**DOI:** 10.3389/fcimb.2022.824788

**Published:** 2022-01-28

**Authors:** Aaron D. Gingerich, Jarrod J. Mousa

**Affiliations:** ^1^ Center for Vaccines and Immunology, College of Veterinary Medicine, University of Georgia, Athens, GA, United States; ^2^ Department of Infectious Diseases, College of Veterinary Medicine, University of Georgia, Athens, GA, United States; ^3^ Department of Biochemistry and Molecular Biology, Franklin College of Arts and Sciences, University of Georgia, Athens, GA, United States

**Keywords:** *Streptococcus pneumoniae*, monoclonal antibody, opsonophagocytic, immune evasion, pneumococcal vaccination

## Abstract

The gram-positive bacterium *Streptococcus pneumoniae* is a leading cause of pneumonia, otitis media, septicemia, and meningitis in children and adults. Current prevention and treatment efforts are primarily pneumococcal conjugate vaccines that target the bacterial capsule polysaccharide, as well as antibiotics for pathogen clearance. While these methods have been enormously effective at disease prevention and treatment, there has been an emergence of non-vaccine serotypes, termed serotype replacement, and increasing antibiotic resistance among these serotypes. To combat *S. pneumoniae*, the immune system must deploy an arsenal of antimicrobial functions. However, *S. pneumoniae* has evolved a repertoire of evasion techniques and is able to modulate the host immune system. Antibodies are a key component of pneumococcal immunity, targeting both the capsule polysaccharide and protein antigens on the surface of the bacterium. These antibodies have been shown to play a variety of roles including increasing opsonophagocytic activity, enzymatic and toxin neutralization, reducing bacterial adherence, and altering bacterial gene expression. In this review, we describe targets of anti-pneumococcal antibodies and describe antibody functions and effectiveness against *S. pneumoniae*.

## Introduction

*Streptococcus pneumoniae* is a gram-positive opportunistic bacterial pathogen that colonizes the upper respiratory tract, and is a leading cause of bacterial infections worldwide (Weiser et al., 2018), (Denny and Loda, 1986). Bacterial colonization is a precursor to pneumococcal disease, which can manifest as otitis media, pneumonia, septicemia and meningitis. *S. pneumoniae* is found in up to 27-65% of children and <10% of adults in a commensal carriage state (Yahiaoui et al., 2016). In 2000, it was estimated that *S. pneumoniae* was responsible for over 800,000 deaths in children annually (O’Brien et al., 2009). Due to the continued high burden of disease despite an effective vaccine, *S. pneumoniae* was designated a priority pathogen by the World Health Organization in 2017 (WHO, 2017). Multiple colonization events in the lifetime of an individual result in serum antibody responses to the capsular polysaccharide (CPS) (Weinberger et al., 2008) and protein antigens (McCool et al., 2002; Zhang et al., 2006; Prevaes et al., 2012; Turner et al., 2013). The CPS is a critical virulence factor for *S. pneumoniae* survival during invasive disease, and the CPS can inhibit the innate immune response *via* inhibition of phagocytosis, preventing recognition of the bacteria by host receptors, and evasion of neutrophil extracellular traps (Hyams et al., 2010). Each pneumococcal serotype, of which 100 serotypes have been identified, has a distinct CPS that varies in its biochemical and antigenic structure (Ganaie et al., 2020). The most effective preventative measure against pneumococcal infection is vaccination with either a 23-valent pneumococcal polysaccharide vaccine (PPSV23) or a multivalent pneumococcal conjugate vaccine (PCV7, PCV10, PCV13, PCV15, or PCV20). While the CPS is the primary antigen in both vaccines, the CPS antigens in PCV-based vaccines are linked to a carrier protein to induce T-dependent antibody responses leveraging the hapten-carrier effect. However, due to the limited serotypes included in PCVs, protection is incomplete, and this has caused an increase of non-vaccine serotypes in carriage and disease (Klugman, 2009; von Gottberg et al., 2014). Nonencapsulated strains of *S. pneumoniae* are also able to colonize the nasopharyngeal tract and are not affected by current vaccines (Keller et al., 2016). These strains have unique surface proteins that allow for colonization and virulence in the absence of the CPS (Keller et al., 2016).

**Figure 1:**
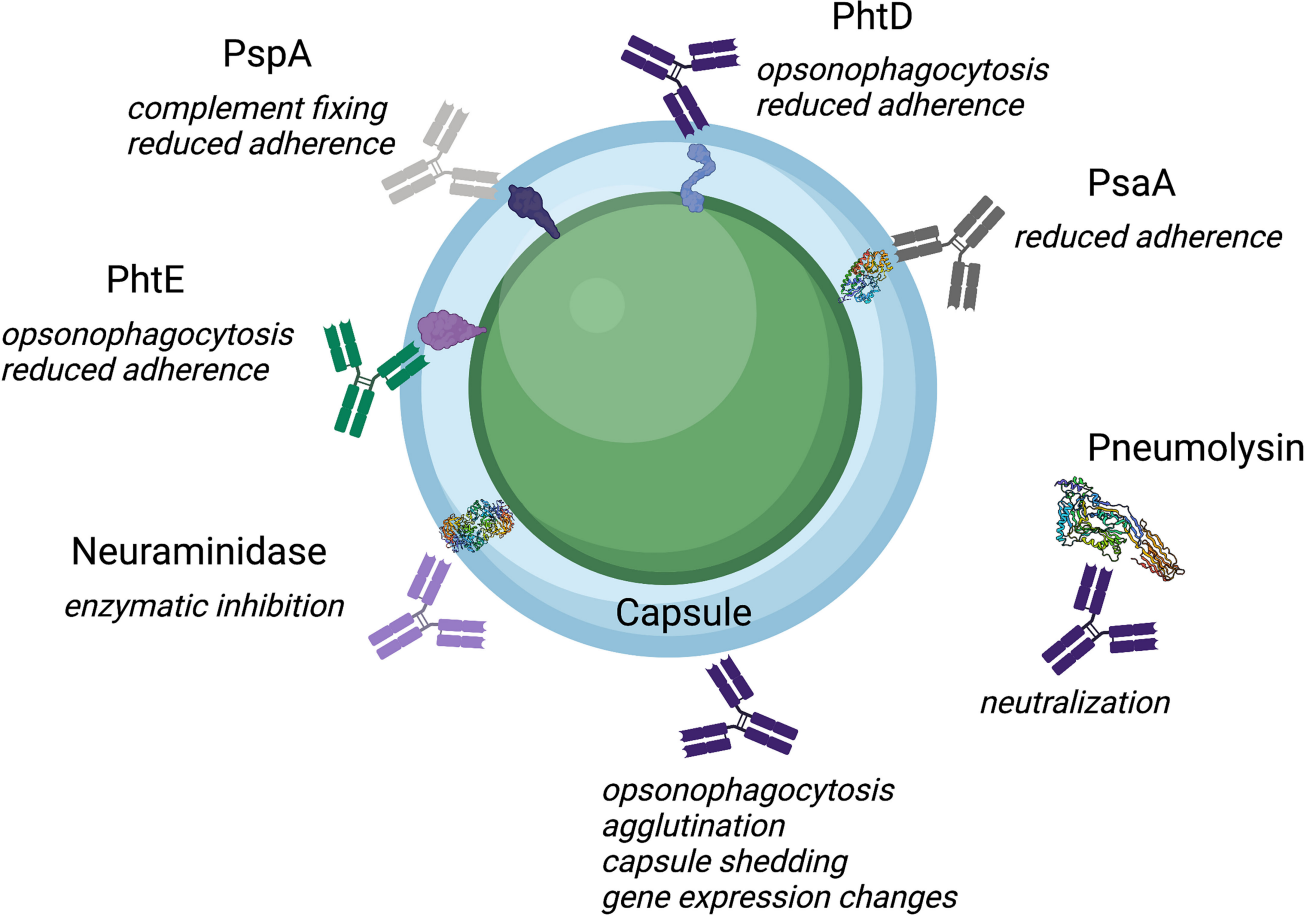
Illustration of the diverse mechanisms and targets of anti-pneumococcal antibodies. Protein antigens are displayed within the capsule as cartoons or as protein models when available from the protein data bank.

In light of limited serotype coverage, increasing prevalence of non-vaccine serotypes, and increasing antibiotic resistance among non-vaccine serotypes, conserved protein antigens and other molecules have also been studied as potential vaccine candidates, as these would presumably induce broadly reactive antibodies that would be effective against a greater number of serotypes than CPS-based vaccines (Daniels et al., 2016). Multiple antigens have been tested in animal models and clinical trials, including the toxin pneumolysin (Ply) (Kamtchoua et al., 2013; Odutola et al., 2017; Hammitt et al., 2019), pneumococcal surface protein A (PspA) (Briles et al., 2000b; Nabors et al., 2000; Frey et al., 2013), pneumococcal surface adhesin A (PsaA) (Wang et al., 2010; Moffitt and Malley, 2016), pneumococcal choline binding protein A (PcpA) (Glover et al., 2008; Verhoeven et al., 2014; Xu et al., 2017), pneumococcal histidine triad protein (PhtD) (Denoël et al., 2011b; Kaur et al., 2014; André et al., 2021), Phosphorycholine (PC) (Trolle et al., 2000; Tanaka et al., 2007), Neuraminidase (Nan) (Janesch et al., 2018) and choline binding proteins (CbpA,CbpG) (Mann et al., 2006; Hernani et al., 2011; Ricci et al., 2011). Antibodies are a key component of pneumococcal immunity, and their mechanisms of action are important to understand for vaccine design efforts (**Figure 1** and **Table 1**). Here we review anti-pneumococcal antibodies, including antibody targets and known mechanisms of action.

**Table 1:** Antigens evaluated in clinical trials and animal models.

Antigen	Antigen function	Antibody function	Tested as Vaccine?	Species tested	Vaccine status	Protection	Antibody Therapy	Protection	
Capsular polysaccharide	Inhibition of phagocytosis	Opsonophagocytosis	Yes	Murine	FDA approved	Yes	Yes	Yes	Burns et al., 2003; Burns et al., 2005; Fabrizio et al., 2007; Tian et al., 2009; Yano et al., 2011; Sarah et al., 2012; Doyle and Pirofski, 2016; Keller et al., 2016; Mitsi et al., 2017; Babb et al., 2021; Doyle et al., 2021
	Blocking host receptors	Agglutination		Human					
	Evasion of NETs	Capsule shedding							
		Gene expression							
Phosphorylcholine	Bacterial adherence	Reduced adherence	Yes	Murine	Murine model	Yes	Yes	Yes	Wallick et al., 1983; Briles et al., 1992; Fischer et al., 1995; Trolle et al., 2000; Tanaka et al., 2007
PspA	Inhibition of complement deposition	Increase C3 deposition	Yes	Murine	Phase 1	Protection not yet assessed in humans. Yes, in animal models	Yes	Yes	Wu et al., 1997; Briles et al., 2000b; Briles et al., 2000c; Nabors et al., 2000; Ferreira et al., 2006b; Daniels et al., 2010; Bitsaktsis et al., 2012; Frey et al., 2013; Piao et al., 2014; Nagano et al., 2018; Nakahashi-Ouchida et al., 2021
	Binding lactoferrin	Inhibition of lactoferrin binding		Human					
	Evasion of NETs	Enhancing NETs							
PsaA	ABC transporter of Zn2+ and Mn2+	Reduced adherence	Yes	Murine	Phase 1	Protection not yet assessed in humans. Partial protection in animal models	ND	ND	Talkington et al., 1996; Briles et al., 2000a; Miyaji et al., 2001; Romero-Steiner et al., 2003; Oliveira et al., 2006; Pimenta et al., 2006; Romero-Steiner et al., 2006; Wang et al., 2010
	Attachment to epithelial cells			Human					
	Protection against oxidative stress								
PhtD	Inhibition of complement deposition	Increase C3 deposition	Yes	Murine	Phase1/2	Yes, in animal models. Did not reduce colonization or otitis media in humans	Yes	Yes	Wizemann et al., 2001; Denoël et al., 2011a; Denoël et al., 2011b; Godfroid et al., 2011; Bologa et al., 2012; Khan and Pichichero, 2012; Seiberling et al., 2012; Plumptre et al., 2013b; Ravinder et al., 2014; Berglund et al., 2014; Leroux-Roels et al., 2014; Odutola et al., 2017; Papastamatiou et al., 2018; Visan et al., 2018; Hammitt et al., 2019; André et al., 2020; Huang et al., 2021
	Zn^2+^ acquisition	Reduced adherence		Macaque					
	Adherance to epithelial cells	Opsonophagocytosis		Human					
Neuraminidase	Sugar acquisition	Enzymatic inhibition	No	ND	ND	ND	Yes	No	Janesch et al., 2018
	Inhibition of complement deposition								
	Adherence to endothelial cells								
	Promotes biofilm formation								
Pneumolysin	Cytotoxic activity	Neutralization	Yes	Murine	Phase1/2	Yes in animal models. Did not reduce colonization or otitis media in humans	Yes	Yes	García-Suárez et al., 2004; Ferreira et al., 2006a; Sanders et al., 2010; Lu et al., 2014; Mann et al., 2014; Hermand et al., 2017; Odutola et al., 2017; Hammitt et al., 2019; Petukhova et al., 2020; Thanawastien et al., 2021
	Inhibition of complement deposition			Macaque					
	Biofilm formation			Human					
CbpG	Cleavage of casein and fibronectin	Opsonophagocytosis	Yes	Murine	Murine model	Yes	Yes	Yes	Mann et al., 2006; Kazemian et al., 2018
PcpA	Adherence to epithelial cells	Increase C3 deposition	Yes	Murine	Phase 1	Protection not yet assessed in humans. Yes, in animal models	Yes	Yes	Glover et al., 2008; Verhoeven et al., 2014; Brooks et al., 2015; Ochs et al., 2016; Xu et al., 2017; Visan et al., 2018
		Reduced adherence		Human					
		Opsonophagocytosis							
PspC/CbpA	Adherence to epithelial cells	Increase C3 deposition	Yes	Murine	Murine model	Protective against certain serotypes	Yes	Protective when used with other antibodies	Cao et al., 2007; Lu et al., 2008; Ferreira et al., 2009; Hernani et al., 2011; Ricci et al., 2011; Mann et al., 2014; Chen et al., 2015
	Binding complement factor H, C4BP, and vitronectin	Block fH binding							
		Opsonophagocytosis							

### Antibodies to the Pneumococcal Capsular Polysaccharide

One of the most important virulence factors of *S. pneumoniae* is the CPS, which encases the bacterium and is the defining antigen for serotype identification. Currently, 100 distinct capsule serotype structures have been identified, with at least 30 serotypes being responsible for invasive pneumococcal disease (Luck et al., 2020). The CPS is typically negatively charged, which allows the bacteria to avoid mucous entrapment and enables colonization of the nasopharynx (Magee and Yother, 2001; Nelson et al., 2007; Li et al., 2013). As the CPS encases the bacterium, it protects deeper antigenic structures, and inhibits the binding of immunoglobulins, complement components, and C-reactive protein (Weiser et al., 2018). The CPS also reduces opsonization with C3b/iC3b, thereby impairing the receptors of phagocytic cells from interacting with antibody Fc regions (Abeyta et al., 2003; Hyams et al., 2010). It has been hypothesized that the CPS also protects bacteria from entrapment by neutrophil extracellular traps (Wartha et al., 2007). Due to the vital importance of the CPS to bacterial survival and virulence, it has long been a therapeutic target. PPSV23 and PCV vaccines have been approved for use in humans and have been widely effective. However, there has been a rise in the incidence of nonvaccine serotypes, and serotypes 3 and 19A have also persisted despite vaccination efforts (Wantuch and Avci, 2018; Linley et al., 2019).

**Figure 2:**
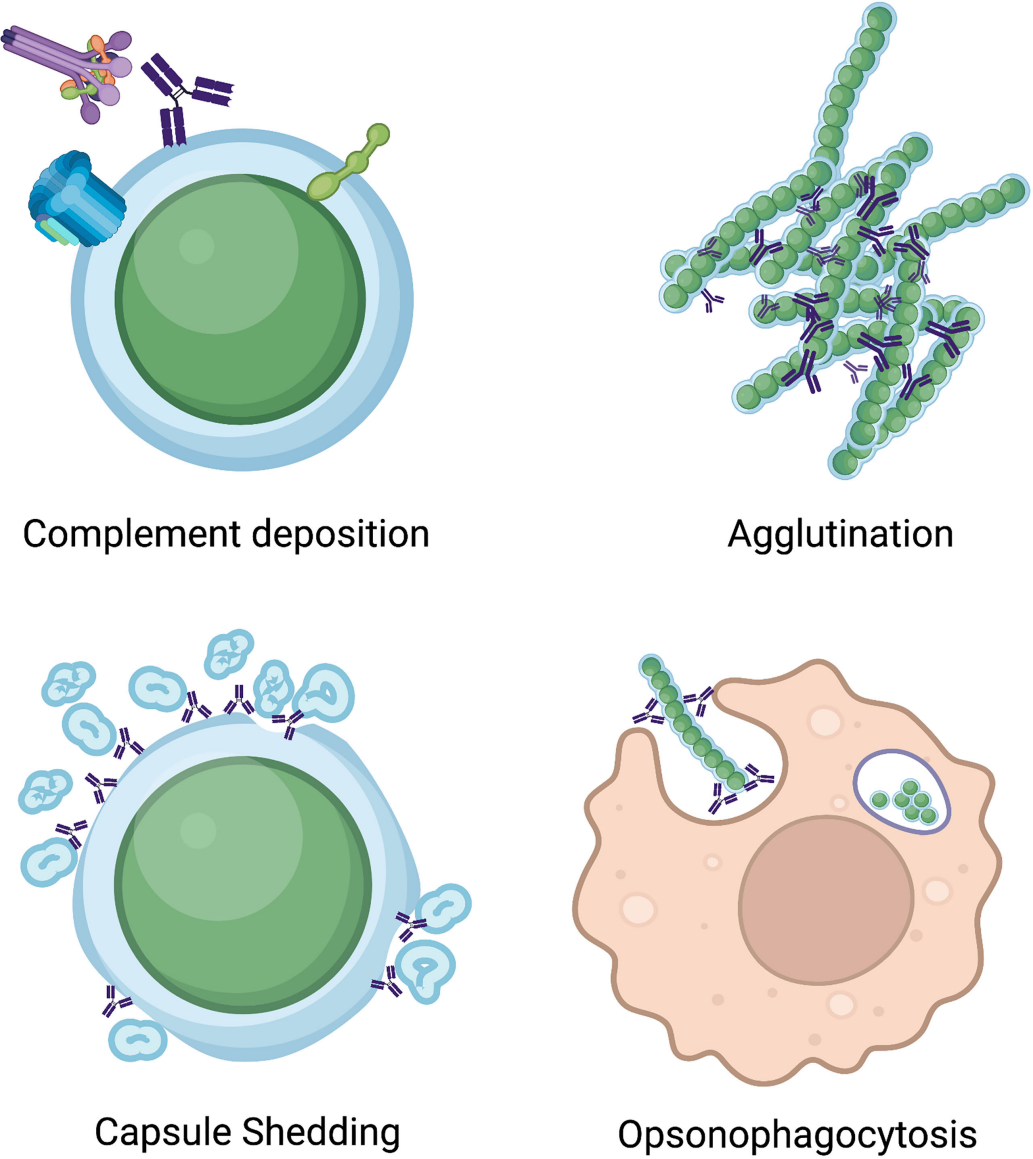
Illustration of mechanisms of anti-pneumococcal antibodies. Antibodies binding pneumococcal antigens can increase complement deposition, leading to formation of the membrane attack complex, induce bacterial agglutination, cause capsule shedding, and increase opsonophagocytic activity of phagocytic cells.

A key function of CPS targeting vaccines is to elicit antibodies that have opsonophagocytic activity, which has been well defined to be a predictor of protection (Poehling et al., 2006; Ekström et al., 2007; Goldblatt et al., 2010; Prymula et al., 2011; Dagan et al., 2013; Lee et al., 2014) (**Figure 2**). Indeed, *in vitro* opsonophagocytic assays are the gold standard for measuring antibody-mediated immunity (Romero-Steiner et al., 1997; Song et al., 2013; Toh et al., 2021). In addition, antibody agglutination activity is a correlate of protection (Bull, 1915), as the establishment of colonization by *S. pneumoniae* is a critical first step in disease pathogenesis, and antibody agglutinating activity inhibits colonization (Bogaert et al., 2004; Simell et al., 2012) (**Figure 2**). For example, CPS-specific antibodies from nasal washes of vaccinated individuals are important for protection against colonization (Mitsi et al., 2017), and human mAbs targeting the CPS can have both opsonophagocytic and agglutination activity *in vitro* (Babb et al., 2021).

While a majority of described antibodies targeting the CPS rely on increasing opsonophagocytic and agglutinating activity to offer protection, nonopsonic antibodies to the pneumococcal CPS can also offer protection. The first study to discover this phenomenon found that a mouse IgG_1_, mAb 1E2, targeting the serotype 3 CPS was unable to opsonize and kill bacteria *in vitro* yet was protective *in vivo* (Tian et al., 2009). In several follow up studies, mAb 1E2 was shown to alter the expression of over 50 genes (Doyle et al., 2021), which included genes involved in quorum sensing and increased fratricide (Yano et al., 2011). Upon interaction with mAb 1E2, iron uptake (*piuB* gene) was increased, sensitivity to oxidative stress (*dpr* gene) was amplified, and rapid capsule shedding was observed (Doyle et al., 2021) (**Figure 2**). mAb 1E2 also reduces colonization in mice (Doyle and Pirofski, 2016). A mechanistic study on mAb 1E2 revealed that neutrophils were not needed for its protective efficacy (Tian et al., 2009), but macrophages were required (Sarah et al., 2012). These studies demonstrate that anti-CPS antibody functions beyond opsonophagocytic and agglutination activity can protect against pneumococcal disease, and such functions may be important to consider in current and future vaccines.

Serotype specific IgG targeting the CPS is sufficient to protect against the homologous serotype, and additional antibody isotypes beyond IgG can offer protection. Serotype 8 specific mAbs NAD (an IgA) and D11 (an IgM) have been examined *in vitro*, and NAD was shown to increase complement deposition (**Figure 2**) whereas D11 was not, and neither NAD nor D11 promoted significant neutrophil mediated killing in an opsonophagocytic assay (Burns et al., 2003). In the presence of bacteria, complement, and D11 or NAD, a decrease in IL-8 secretion by neutrophils was observed (Burns et al., 2003). Both the D11 and NAD antibodies were found to be protective against infection in the mouse model (Burns et al., 2003). Additional studies with D11 revealed treated mice had significantly less IFN-γ, MIP-2, IL-12, MCP-1/JE, and TNF-α compared to control mice (Burns et al., 2005). mAb A7, a serotype 3-specific IgM, induced a decrease in keratinocyte-derived chemokine, IL-6 and macrophage inflammatory protein-2 in mAb treated mice, similar to levels seen in penicillin treated mice (Fabrizio et al., 2007). Together these studies demonstrate that the connection between antibody-mediated protection and immunomodulation plays a key role in protection *in vivo* and should be further explored.

### Antibodies to Pneumococcal Proteins

#### Pneumococcal Surface Protein A

While vaccine-induced CPS-specific antibodies can protect against colonization with vaccine-included serotypes, natural colonization leads the induction of both CPS-specific antibodies and anti-protein antibodies (Wilson et al., 2017). Pneumococcal surface protein A (PspA) is one of the most prevalent antigens on the surface of *S. pneumoniae* and plays a major role in protective immunity (Khan and Jan, 2017). PspA aids the bacteria in evading the bactericidal activity of neutrophil extracellular traps (Martinez et al., 2019), inhibits complement (Tu et al., 1999; Ren et al., 2004; Ren et al., 2012) by reducing the amount of C3 that is deposited on the bacteria (Mukerji et al., 2012), and binds lactoferrin, which likely blocks the active site of apolactoferrin responsible for bacterial killing (Shaper et al., 2004; Bitsaktsis et al., 2012; Mirza et al., 2016). PspA is classified into 3 families with 6 clades based on sequence similarities in the variable N-terminal α-helical region (Hollingshead et al., 2000). Numerous studies have demonstrated the protective effects of PspA-based vaccines, which lead to increased survival and decreased bacterial burden in animal models (Wu et al., 1997; Briles et al., 2000c; Ferreira et al., 2006b; Daniels et al., 2010; Piao et al., 2014; Nagano et al., 2018; Nakahashi-Ouchida et al., 2021). It has also been demonstrated that antibodies targeting PspA can have their function enhanced *via* targeting activating FcγRI with fusion proteins consisting of PspA and IgG2a Fc, which then is able to enhance PspA-specific immune responses (Wiedinger et al., 2020). Another study demonstrated that by fusing a humanized single-chain antibody component (in which the variable domain binds to FcγRI) to PspA, protection could be achieved in the absence of adjuvant (Bitsaktsis et al., 2012). This vaccine could be enhanced further by adding an additional FcγRI binding moiety to the vaccine mentioned above (Kumar et al., 2020). PspA vaccines have advanced to human clinical trials and demonstrated safety and robust antibody responses (Briles et al., 2000b; Nabors et al., 2000; Frey et al., 2013), however, concerns exist about using full length PspA due to its sequence homology with human cardiac myosin (Ginsburg et al., 2012).

Vaccine derived antibodies targeting PspA mainly function by increasing complement C3 deposition leading to increased phagocytosis. Passive immunization with an anti-PspA mAb XiR278 protected mice from infection at 10x the LD_50_ when given before or shortly after pneumococcal infection (Swiatlo et al., 2003). Another study generated broadly reactive anti-PspA mouse mAbs, one of which, mAb 140H1, bound to 98% of the 48 strains tested, which encompasses the clinically relevant PspA clades 1-5 (Kristian et al., 2016). mAb 140H1 was shown to be protective in *in vivo* mouse sepsis and pneumonia models, including 24 hrs after intranasal infection (Kristian et al., 2016). This study also demonstrated that mAb 140H1 and others were able to increase C3 deposition on the surface of bacteria and increase bacterial killing in opsonophagocytic killing assays. This suggests that the complement deposition induced by these anti-PspA mAbs facilitates phagocytic uptake and killing by neutrophils (Kristian et al., 2016). In support of this observation, increased C3 deposition on the surface of bacteria *via* anti-PspA mAb binding has been demonstrated in numerous studies (Ren et al., 2004; Ren et al., 2012; Bitsaktsis et al., 2012; Goulart et al., 2013; Khan et al., 2015; Genschmer et al., 2019; Wiedinger et al., 2020). Additional functions for anti-PspA mAbs include inhibition of lactoferrin by anti-PspA mAbs (Bitsaktsis et al., 2012), and enhanced trapping and killing of *S. pneumoniae* by neutrophil extracellular traps (Martinez et al., 2019).

#### Pneumococcal Surface Adhesin A

The pneumococcal surface adhesin A (PsaA) is a multifunctional surface exposed lipoprotein that is found in all known serotypes (Sampson et al., 1997). PsaA is an ABC transporter binding protein that is capable of transporting Zn^2+^ and Mn^2+^ (Dintilhac et al., 1997; Lawrence et al., 1998; Jakubovics and Jenkinson, 2001; W et al., 2004). PsaA can also facilitate attachment to host cells (Sampson et al., 1994; Novak et al., 2000; Romero-Steiner et al., 2003; McAllister et al., 2004; Romero-Steiner et al., 2006) and protect against oxidative stress (Tseng et al., 2002). The binding receptor for PsaA is E-cadherin, the cell-cell junction protein found in respiratory epithelial cells (Anderton et al., 2007). PsaA employs multiple effects on host cells including cytoplasmic foaming, excessive vacuolation, and nuclear structure changes leading to colonization and internalization of the bacteria (Anderton et al., 2007). It was recently discovered that PsaA interacts with human Annexin A2 (ANXA2) (Hu et al., 2021). Immunization studies with recombinant PsaA showed a significant reduction in recovery time against pneumococcal carriage (Briles et al., 2000a; Miyaji et al., 2001; Oliveira et al., 2006; Pimenta et al., 2006; Wang et al., 2010). However when used in a systemic challenge model those protective effects were either minimal (Talkington et al., 1996) or not significant (Wang et al., 2010). Anti-PsaA antibodies can reduce the ability of *Streptococcus pneumoniae* to bind to Detroit 562 cells (pharyngeal epithelial cells) *in vitro* (Romero-Steiner et al., 2003; Romero-Steiner et al., 2006).

#### Pneumococcal Histidine Triad Proteins

The pneumococcal histidine triad proteins are a group of four proteins (PhtA, PhtB, PhtD, PhtE) that are present on the surface of *S. pneumoniae* (Adamou et al., 2001). PhtD is the most conserved of the group with 91-98% identity across strains isolated from cases of invasive disease (Yun et al., 2015). While the function of PhtD is not fully understood, it is important for attachment to respiratory epithelial cells *in vitro* (Khan and Pichichero, 2012; Plumptre et al., 2013a; Kallio et al., 2014). Additionally, PhtD has been shown *in vitro* to aid in Zn^2+^ acquisition and ultimately bacterial homeostasis as Zn^2+^ is a vital nutrient (Ogunniyi et al., 2009; Eijkelkamp et al., 2016). PhtD can also reduce complement deposition *via* Factor H (FH) (Ogunniyi et al., 2009). Several studies have demonstrated that immunization with PhtD or other Pht antigens elicits antibodies that protect against sepsis, pneumonia, and reduced colonization in animal models (Wizemann et al., 2001; Denoël et al., 2011a; Denoël et al., 2011b; Godfroid et al., 2011; Plumptre et al., 2013b; Ravinder et al., 2014; Papastamatiou et al., 2018; André et al., 2020). In a non-human primate model, vaccination with a combined PhtD/pneumolysin formulation was protective against lethal challenge (Denoël et al., 2011b). PhtD-based vaccine candidates have advanced to clinical trials, and several trials have demonstrated that immunization with PhtD was well tolerated and immunogenic (Bologa et al., 2012; Khan and Pichichero, 2012; Seiberling et al., 2012; Berglund et al., 2014; Leroux-Roels et al., 2014; Hammitt et al., 2019). However in a clinical trial in 6-12 month old infants, vaccination with PhtD/dPly/PCV13 did not show an increase in vaccine efficacy against acute otitis media compared to the standard PCV13 vaccine (Hammitt et al., 2019). In an additional study, a PHiD-CV/dPly/PhtD vaccine containing a 10-valent polysaccharide conjugate (10VT) combined with pneumolysin toxoid and pneumococcal histidine triad protein D was immunogenic but no improvement in pneumococcal carriage in infants was seen regardless of dose or schedule (Odutola et al., 2017). Vaccine induced PhtD antibodies were shown to increase complement C3b deposition on the bacterial surface and increase phagocytosis.

Antibodies targeting PhtD have demonstrated protection in preclinical animal models. Anti-PhtD polyclonal antibodies are able to reduce colonization (Ravinder et al., 2014) and protect against lethal sepsis (Brookes et al., 2015) in a passive transfer mouse model. The use of anti-PhtD mAbs has also been explored. Human anti-PhtD mAbs are protective against both sepsis and pneumonia models of infection in mice when given prior to infection (Huang et al., 2021). Additionally, administration of an anti-PhtD mAb (PhtD3) 24 hours post infection was able to rescue mice from infection (Huang et al., 2021). An *in vivo* mechanism for anti-PhtD antibodies has not been fully elucidated, however, a recent study demonstrated that when depleting complement *via* cobra venom factor the protective effect of anti-PhtD antibodies was lost (Visan et al., 2018). Furthermore, depletion of macrophages but not neutrophils also resulted in loss of protection (Visan et al., 2018). The mechanism of anti-PhtD antibodies has been tested *in vitro* where it was observed that antibodies are able to inhibit bacterial attachment to epithelial cells (Kallio et al., 2014; Kaur et al., 2014), promote complement deposition (André et al., 2021) (Visan et al., 2018), and increase bacterial phagocytosis (Visan et al., 2018; Malekan et al., 2020; André et al., 2021; Huang et al., 2021).

#### Neuraminidase

*S. pneumoniae* utilizes neuraminidases for sugar acquisition, and these neuraminidases are a key virulence factor of which three have been described: NanA, NanB and NanC (Cámara et al., 1994; Berry et al., 1996; Xu et al., 2011; Hammond et al., 2021). These neuraminidases cleave terminal sialic acids providing essential nutrients (King et al., 2006; Burnaugh et al., 2008), interfere with C3 deposition (Dalia et al., 2010), promote biofilm formation (Parker et al., 2009), and are required for adherence and invasion of brain endothelial cells (Uchiyama et al., 2009). Due to the multitude of essential functions for *S. pneumoniae* these neuraminidases provide an attractive target for therapeutic antibodies. Utilizing an *in vitro* assay with differentiated airway epithelial cells, it was shown that anti-neuraminidase mAbs can preserve the terminal epithelial sugar composition during *S. pneumoniae* infection leading to a 10-20 fold decrease in bacterial growth compared to control mAbs (Janesch et al., 2018). Similar effects were seen in an *in vivo* mouse model, where anti-neuraminidase mAbs led to a reduction in desialylation of the airways (Janesch et al., 2018). However, in an acute murine pneumonia treatment with the anti-neuraminidase mAbs there was no effect on survival, lung burden, or host inflammatory responses (Janesch et al., 2018). While anti-neuraminidase mAbs exert an inhibitory effect on the neuraminidases, this does not lead to a positive effect on mortality or bacterial burden in a murine model of acute pneumonia (Janesch et al., 2018).

#### Pneumolysin

The virulence factor pneumolysin (Ply) is a cholesterol-dependent cytolysin that plays an essential role in pneumococcal disease. Pneumolysin lacks a secretory signal and is localized in the cytoplasm or the cell wall, and is released during autolysis and bacterial growth (García-Suárez et al., 2007; Price and Camilli, 2009). Pneumolysin exhibits a wide range of effects, including cytotoxic activity (Steinfort et al., 1989; Rubins et al., 1992; Rayner et al., 1995) to host cells, increasing bacterial penetration (Ring et al., 1998; Zysk et al., 2001), increasing inflammation (Feldman et al., 1991; Cockeran et al., 2001; Yoo et al., 2010; Subramanian et al., 2019), blocking complement (Paton et al., 1984; Mitchell et al., 1991; Rubins et al., 1995; Alcantara et al., 2001), and being a vital factor in biofilm formation and pneumococcal transmission (Shak et al., 2013; Marks et al., 2014; Zafar et al., 2017). Due to its conserved nature between strains and serotypes (Han and Zhang, 2019), (Kanclerski and Möllby, 1987) pneumolysin has been well studied as a therapeutic target. Numerous studies investigating antibodies targeting Ply have demonstrated their protective efficacy. Ply renders platelets nonfunctional and inhibits platelet-thrombus formation in whole blood, and an *in vitro* study demonstrated that anti-Ply antibodies are able to neutralize Ply and rescue platelet function (Jahn et al., 2020). Murine derived anti-pneumolysin mAbs were able to neutralize its cytolytic activity and inhibit binding of pneumolysin to cholesterol (Kucinskaite-Kodze et al., 2020). In a recent study, anti-pneumolysin human polyclonal antibodies did not decrease adherence of TIGR4 to A549 cells *in vitro*, however, in an *in vivo* murine model, passive transfer of anti-pneumolysin antibodies did show a significant decrease in colonization of TIGR4 (Kaur et al., 2014). Biofilm formation plays a key role in pneumococcal colonization and Ply is important for biofilm formation, independent of its cytolytic activity (Shak et al., 2013). High serum levels of anti-Ply antibodies have been correlated to delayed colonization in infants (Holmlund et al., 2006; Francis et al., 2009), and low anti-Ply antibodies may be a predisposing factor in developing pneumococcal pneumonia (Huo et al., 2004). Inactive Ply mutants are able to induce antibodies that neutralize Ply *via* an *in vitro* anti-hemolytic assay (Kamtchoua et al., 2013). Several studies have demonstrated that immunization with Ply generates anti-pneumolysin antibodies that are protective (Sanders et al., 2010; Lu et al., 2014; Mann et al., 2014; Hermand et al., 2017; Petukhova et al., 2020; Thanawastien et al., 2021). The direct use of anti-pneumolysin mAbs was protective when administered intravenously in a lethal intranasal model of infection in mice (García-Suárez et al., 2004). However, a recent study showed that using a DNA vaccine vector, low levels of anti-pneumolysin antibodies were generated that were not protective in a septic challenge model in mice (Ferreira et al., 2006a). In human clinical trials, anti-pneumolysin antibodies derived from vaccination are able to neutralize pneumolysin (Kamtchoua et al., 2013) but no differences were seen in colonization, bacterial load, or clearance due to the vaccine compared to controls (Odutola et al., 2017; Hammitt et al., 2019). While antibodies targeting Ply have been shown to function through the neutralization of its cytolytic activity, antibody inhibition of other Ply functions such as adherence, complement blocking, and biofilm formation have not been demonstrated.

#### Choline Binding Proteins

The virulence factor CbpG is an adhesin with putative serine protease activity in both colonization and sepsis (Galán-Bartual et al., 2015). The protease portion of CbpG is able to cleave casein and fibronectin, and enzymatic activity is able to remain intact regardless of being surface bound or secreted (Mann et al., 2006). Another closely related antigen, CbpM, was demonstrated to bind to fibronectin facilitating *S. pneumoniae* attachment to epithelial cells (Afshar et al., 2016). Vaccination with recombinant CbpG induces antibodies that are able to confer protection against colonization to a limited degree and provide robust protection against systemic infection (Mann et al., 2006). In another study, mice immunized with CpbG or CbpM and challenged *via* intraperitoneal injection had a significant increase in protection and clearance of bacteria as early as 48 hours post infection (Kazemian et al., 2018). Serum containing anti-CbpG and anti-CbpM antibodies from the aforementioned study were also found to increase neutrophil mediated opsonophagocytosis (Kazemian et al., 2018). Finally, a passive transfer model using anti-CbpG and anti-CbpM antibodies in mice demonstrated a protective effect against *S. pneumoniae* challenge although not as robust as that seen in immunization studies with the antigens (Kazemian et al., 2018). Currently, antibodies targeting CbpG and CbpM have been shown to increase neutrophil mediated opsonphagocytosis, but other functions of these antibodies need to be further elucidated.

PcpA is another member of the choline binding protein family that is under the control of the manganese-dependent regulator *psaR*, and high concentrations of Mn suppress expression (Johnston et al., 2006). Immunization with PcpA elicits antibody responses that provide protection against lung and systemic infections (Glover et al., 2008; Verhoeven et al., 2014; Xu et al., 2017), but do not protect against colonization in the nasopharynx (Sánchez-Beato et al., 1998). In phase 1 human trials PcpA was used in a trivalent recombinant vaccine containing PhtD, PlyD1 and PcpA, the vaccine demonstrated immunogenicity with an increase in antibody concentration, however protection was not assessed (Brooks et al., 2015). Passive transfer of human anti-PcpA polyclonal antibodies in a murine challenge model were able to mediate protection (Ochs et al., 2016). The adherence effect of PcpA on the surface of bacteria was successfully blocked by utilizing Fab fragments targeting PcpA, which blocked adherence of *S. pneumoniae* to human epithelial cells *in vitro* (Khan et al., 2012). Macrophages but not neutrophils were required for anti-PcpA antibody protective efficacy in a passive transfer model (Visan et al., 2018). Additionally, anti-PcpA antibodies are able to enhance compliment C3b deposition (Visan et al., 2018), increase phagocytosis and block adherence of the bacteria.

CbpA/PspC is involved in the adhesion and colonization of the nasopharynx and in the binding of pIgR (Kerr et al., 2006; Orihuela et al., 2009). PspC is important in the recruitment and binding of complement factor H (Dave et al., 2001; Hammerschmidt et al., 2007; Ricci et al., 2011), evading complement by binding of C4BP (Dieudonné-Vatran et al., 2009; Haleem et al., 2019), and prevention of terminal complement complex mediated lysis by binding vitronectin (Voss et al., 2013; Kohler et al., 2015). Due to its role in virulence on multiple fronts, it is a key immunogenic target, however, while it is present in all clinically relevant serotypes, it is quite variable making it a difficult target for vaccination efforts (Brooks-Walter et al., 1999; Iannelli et al., 2002; Georgieva et al., 2018). This high degree of variability has led to mixed results when PspC has been used for immunization. Studies have demonstrated that immunization with PspC alone (Hernani et al., 2011; Ricci et al., 2011) or a multivalent approach with other pneumococcal antigens PspA and/or Ply are protective (Cao et al., 2007; Mann et al., 2014; Chen et al., 2015). However, other studies have shown that immunization with PspC is not protective (Lu et al., 2008; Ferreira et al., 2009; Ricci et al., 2011). These different results are likely due to the high variability of PspC between different serotypes. Passive immunization with anti-PspC antibodies has demonstrated protection (Mann et al., 2014), however, once again mixed results have been seen where passive immunization with anti-PspC antibodies alone were not protective but showed a protective and synergistic effect once administered with anti-PspA and anti-ClbP antibodies (Cao et al., 2007). PspC antibodies can increase complement deposition (Ricci et al., 2011), interfere with fH binding (Ricci et al., 2011; Glennie et al., 2016) and promote opsonophagocytic killing of *S. pneumoniae* (Ricci et al., 2011; Georgieva et al., 2018) *in vitro*.

### Other Antigens

#### Phosphorylcholine

*S. pneumoniae* contains phosphorylcholine (PC) also known as ChoP, a structural component that is linked to bacterial adherence *via* the platelet activating factor receptor (PAF-R) (Cundell et al., 1995; Iuchi et al., 2019). Several key virulence proteins such as the CBPs and PspA are attached to the cell wall *via* PC (Rosenow et al., 1997). PC-dependent binding to the epithelial receptor asialo-GM1 has also been demonstrated (Sundberg-Kövamees et al., 1996). PC expression is essential to the bacteria as mutant bacteria that do not express PC are unable to colonize the upper respiratory tract in mice and are less virulent in murine sepsis models (Kharat and Tomasz, 2006). The C-reactive protein (CRP) recognizes PC and initiates the classical complement pathway increasing phagocytosis of *S. pneumoniae* (Mold et al., 1982). Due to PCs important role in adherence and association with key proteins, it offers a promising therapeutic target. Early on in the field it was demonstrated that anti-PC antibodies in normal mouse serum provide protection against intravenous pneumococcal challenge (Briles et al., 1981). Anti-PC mAbs were also found to be protective against several different serotypes in a murine infection model (Briles et al., 1992). One study demonstrated that pretreating *S. pneumoniae* with an anti-PC mAb reduced the adherence of *S. pneumoniae* with high levels of PC but not low levels of PC in both *in vitro* and *in vivo* models (Iuchi et al., 2019). Several studies have concluded that immunization with PC elicits anti-PC antibodies that are able to enhance clearance of *S. pneumoniae* and provide protection against pneumococcal infection (Wallick et al., 1983; Fischer et al., 1995; Trolle et al., 2000; Tanaka et al., 2007).

## Conclusion

This review has discussed the important role of anti-pneumococcal antibodies in protection against pneumococcal infections. While currently approved vaccines only target the pneumococcal capsule of the bacteria *via* immunization, more recent studies have demonstrated the potential of targeting conserved antigens that help bacteria evade the immune system. The antimicrobial effects of anti-pneumococcal antibodies are wide ranging and impressive, and include an increase in complement deposition, enhanced opsonophagocytic activity, amplified NET and lactoferrin mediated killing, interference with attachment and penetration of host cells, neutralization of cytotoxic proteins, and modulation of the inflammatory response. A greater understanding of how anti-pneumococcal antibodies function is crucial. Studies to date have demonstrated that optimal antibody responses are unlikely to target one antigen but multiple antigens with different functions essential to *S. pneumoniae* fitness and/or survival. This synergistic approach may be our most successful path against an ever-evolving pathogen. The emergence of non-vaccine serotype and associated antibiotic resistance of pneumococcal isolates illustrates the need for vaccines that are capable of eliciting antibodies with greater serotype coverage and/or mAb treatments targeting conserved surface exposed antigens.

## Author Contributions

AG and JM wrote and revised the manuscript. All authors contributed to the article and approved the submitted manuscript.

## Funding

American Lung Association Innovation Award (JJM) K01OD026569 (JJM).

## Conflict of Interest

AG and JM have applied for a provisional patent application covering human monoclonal antibody sequences for prevention and treatment of pneumococcal infection.

The remaining authors declare that the research was conducted in the absence of any commercial or financial relationships that could be construed as a potential conflict of interest.

## Publisher’s Note

All claims expressed in this article are solely those of the authors and do not necessarily represent those of their affiliated organizations, or those of the publisher, the editors and the reviewers. Any product that may be evaluated in this article, or claim that may be made by its manufacturer, is not guaranteed or endorsed by the publisher.

## References

[B1] Abeyta M. Hardy G. G. Yother J. (2003). Genetic Alteration of Capsule Type But Not PspA Type Affects Accessibility of Surface-Bound Complement and Surface Antigens of Streptococcus Pneumoniae. Infect. Immun. 71, 218–225. doi: 10.1128/IAI.71.1.218-225.2003 12496169 PMC143148

[B2] Adamou J. E. Heinrichs J. H. Erwin A. L. Walsh W. Gayle T. Dormitzer M.. (2001). Identification and Characterization of a Novel Family of Pneumococcal Proteins That Are Protective Against Sepsis. Infect. Immun. 69, 949–958. doi: 10.1128/IAI.69.2.949-958.2001 11159990 PMC97974

[B3] Afshar D. Pourmand M. R. Jeddi-Tehrani M. Saboor Yaraghi A. A. Azarsa M. Shokri F. (2016). Fibrinogen and Fibronectin Binding Activity and Immunogenic Nature of Choline Binding Protein M. Iran. J. Public Health 45, 1610–1617. 28053927 PMC5207102

[B4] Alcantara R. B. Preheim L. C. Gentry-Nielsen M. J. (2001). Pneumolysin-Induced Complement Depletion During Experimental Pneumococcal Bacteremia. Infect. Immun. 69, 3569–3575. doi: 10.1128/IAI.69.6.3569-3575.2001 11349015 PMC98338

[B5] Anderton J. M. Rajam G. Romero-Steiner S. Summer S. Kowalczyk A. P. Carlone G. M.. (2007). E-Cadherin is a Receptor for the Common Protein Pneumococcal Surface Adhesin A (PsaA) of Streptococcus Pneumoniae. Microb. Pathog. 42, 225–236. doi: 10.1016/j.micpath.2007.02.003 17412553

[B6] André G. O. Assoni L. Rodriguez D. Leite L. C. C. Dos Santos T. E. P. Ferraz L. F. C.. (2020). Immunization With PhtD Truncated Fragments Reduces Nasopharyngeal Colonization by Streptococcus Pneumoniae. Vaccine 38, 4146–4153. doi: 10.1016/j.vaccine.2020.04.050 32362528

[B7] André G. O. Borges M. T. Assoni L. Ferraz L. F. C. Sakshi P. Adamson P.. (2021). Protective Role of PhtD and its Amino and Carboxyl Fragments Against Pneumococcal Sepsis. Vaccine 39, 3626–3632. doi: 10.1016/j.vaccine.2021.04.068 34045100

[B8] Babb R. Doyle C. R. Pirofski L.-A. (2021). Isolation and Characterization of Human Monoclonal Antibodies to Pneumococcal Capsular Polysaccharide 3. Microbiol. Spectr. 9, e0144621. doi: 10.1128/Spectrum.01446-21 34756090 PMC8579928

[B9] Berglund J. Vink P. Tavares Da Silva F. Lestrate P. Boutriau D. (2014). Safety, Immunogenicity, and Antibody Persistence Following an Investigational Streptococcus Pneumoniae and Haemophilus Influenzae Triple-Protein Vaccine in a Phase 1 Randomized Controlled Study in Healthy Adults. Clin. Vaccine Immunol. 21, 56–65. doi: 10.1128/CVI.00430-13 24173029 PMC3910926

[B10] Berry A. M. Lock R. A. Paton J. C. (1996). Cloning and Characterization of Nanb, a Second Streptococcus Pneumoniae Neuraminidase Gene, and Purification of the NanB Enzyme From Recombinant Escherichia Coli. J. Bacteriol. 178, 4854–4860. doi: 10.1128/jb.178.16.4854-4860.1996 8759848 PMC178267

[B11] Bitsaktsis C. Iglesias B. V. Li Y. Colino J. Snapper C. M. Hollingshead S. K.. (2012). Mucosal Immunization With an Unadjuvanted Vaccine That Targets Streptococcus Pneumoniae PspA to Human Fcγ Receptor Type I Protects Against Pneumococcal Infection Through Complement- and Lactoferrin-Mediated Bactericidal Activity. Infect. Immun. 80, 1166–1180. doi: 10.1128/IAI.05511-11 22158740 PMC3294663

[B12] Bogaert D. De Groot R. Hermans P. W. M. (2004). Streptococcus Pneumoniae Colonisation: The Key to Pneumococcal Disease. Lancet Infect. Dis. 4, 144–154. doi: 10.1016/S1473-3099(04)00938-7 14998500

[B13] Bologa M. Kamtchoua T. Hopfer R. Sheng X. Hicks B. Bixler G.. (2012). Safety and Immunogenicity of Pneumococcal Protein Vaccine Candidates: Monovalent Choline-Binding Protein A (PcpA) Vaccine and Bivalent PcpA-Pneumococcal Histidine Triad Protein D Vaccine. Vaccine 30, 7461–7468. doi: 10.1016/j.vaccine.2012.10.076 23123106

[B14] Briles D. E. Ades E. Paton J. C. Sampson J. S. Carlone G. M. Huebner R. C.. (2000a). Intranasal Immunization of Mice With a Mixture of the Pneumococcal Proteins PsaA and PspA is Highly Protective Against Nasopharyngeal Carriage of Streptococcus Pneumoniae. Infect. Immun. 68, 796–800. doi: 10.1128/IAI.68.2.796-800.2000 10639448 PMC97207

[B15] Briles D. E. Forman C. Crain M. (1992). Mouse Antibody to Phosphocholine can Protect Mice From Infection With Mouse-Virulent Human Isolates of Streptococcus Pneumoniae. Infect. Immun. 60, 1957–1962. doi: 10.1128/iai.60.5.1957-1962.1992 1563788 PMC257101

[B16] Briles D. E. Hollingshead S. K. King J. Swift A. Braun P. A. Park M. K.. (2000b). Immunization of Humans With Recombinant Pneumococcal Surface Protein A (Rpspa) Elicits Antibodies That Passively Protect Mice From Fatal Infection With Streptococcus Pneumoniae Bearing Heterologous PspA. J. Infect. Dis. 182, 1694–1701. doi: 10.1086/317602 11069242

[B17] Briles D. E. Hollingshead S. K. Nabors G. S. Paton J. C. Brooks-Walter A. (2000c). The Potential for Using Protein Vaccines to Protect Against Otitis Media Caused by Streptococcus Pneumoniae. Vaccine 19 Suppl 1, S87–S95. doi: 10.1016/s0264-410x(00)00285-1 11163470

[B18] Briles D. E. Nahm M. Schroer K. Davie J. Baker P. Kearney J.. (1981). Antiphosphocholine Antibodies Found in Normal Mouse Serum are Protective Against Intravenous Infection With Type 3 Streptococcus Pneumoniae. J. Exp. Med. 153, 694–705. doi: 10.1084/jem.153.3.694 7252411 PMC2186108

[B19] Brookes R. H. Ming M. Williams K. Hopfer R. Gurunathan S. Gallichan S.. (2015). Passive Protection of Mice Against Streptococcus Pneumoniae Challenge by Naturally Occurring and Vaccine-Induced Human Anti-PhtD Antibodies. Hum. Vaccin. Immunother. 11, 1836–1839. doi: 10.1080/21645515.2015.1039210 25912273 PMC4514344

[B20] Brooks W. A. Chang L.-J. Sheng X. Hopfer R. (2015). Safety and Immunogenicity of a Trivalent Recombinant PcpA, PhtD, and PlyD1 Pneumococcal Protein Vaccine in Adults, Toddlers, and Infants: A Phase I Randomized Controlled Study. Vaccine 33, 4610–4617. doi: 10.1016/j.vaccine.2015.06.078 26143615

[B21] Brooks-Walter A. Briles D. E. Hollingshead S. K. (1999). The pspC Gene of Streptococcus Pneumoniae Encodes a Polymorphic Protein, PspC, Which Elicits Cross-Reactive Antibodies to PspA and Provides Immunity to Pneumococcal Bacteremia. Infect. Immun. 67, 6533–6542. doi: 10.1128/IAI.67.12.6533-6542.1999 10569772 PMC97064

[B22] Bull C. G. (1915). THE MECHANISM OF THE CURATIVE ACTION OF ANTIPNEUMOCOCCUS SERUM. J. Exp. Med. 22, 457–464. doi: 10.1084/jem.22.4.457 19867929 PMC2125359

[B23] Burnaugh A. M. Frantz L. J. King S. J. (2008). Growth of Streptococcus Pneumoniae on Human Glycoconjugates is Dependent Upon the Sequential Activity of Bacterial Exoglycosidases. J. Bacteriol. 190, 221–230. doi: 10.1128/JB.01251-07 17981977 PMC2223752

[B24] Burns T. Abadi M. Pirofski L.-A. (2005). Modulation of the Lung Inflammatory Response to Serotype 8 Pneumococcal Infection by A Human Immunoglobulin M Monoclonal Antibody to Serotype 8 Capsular Polysaccharide. Infect. Immun. 73, 4530–4538. doi: 10.1128/IAI.73.8.4530-4538.2005 16040964 PMC1201218

[B25] Burns T. Zhong Z. Steinitz M. Pirofski L. (2003). Modulation of Polymorphonuclear Cell Interleukin-8 Secretion by Human Monoclonal Antibodies to Type 8 Pneumococcal Capsular Polysaccharide. Infect. Immun. 71, 6775–6783. doi: 10.1128/IAI.71.12.6775-6783.2003 14638763 PMC308885

[B26] Cámara M. Boulnois G. J. Andrew P. W. Mitchell T. J. (1994). A Neuraminidase From Streptococcus Pneumoniae has the Features of a Surface Protein. Infect. Immun. 62, 3688–3695. doi: 10.1128/iai.62.9.3688-3695.1994 8063384 PMC303019

[B27] Cao J. Chen D. Xu W. Chen T. Xu S. Luo J.. (2007). Enhanced Protection Against Pneumococcal Infection Elicited by Immunization With the Combination of PspA, PspC, and ClpP. Vaccine 25, 4996–5005. doi: 10.1016/j.vaccine.2007.04.069 17524530

[B28] Chen A. Mann B. Gao G. Heath R. King J. Maissoneuve J.. (2015). Multivalent Pneumococcal Protein Vaccines Comprising Pneumolysoid With Epitopes/Fragments of CbpA and/or PspA Elicit Strong and Broad Protection. Clin. Vaccine Immunol. 22, 1079–1089. doi: 10.1128/CVI.00293-15 26245351 PMC4580740

[B29] Cockeran R. Steel H. C. Mitchell T. J. Feldman C. Anderson R. (2001). Pneumolysin Potentiates Production of Prostaglandin E(2) and Leukotriene B(4) by Human Neutrophils. Infect. Immun. 69, 3494–3496. doi: 10.1128/IAI.69.5.3494-3496.2001 11292782 PMC98318

[B30] Cundell D. R. Gerard N. P. Gerard C. Idanpaan-Heikkila I. Tuomanen E. I. (1995). Streptococcus Pneumoniae Anchor to Activated Human Cells by the Receptor for Platelet-Activating Factor. Nature 377, 435–438. doi: 10.1038/377435a0 7566121

[B31] Dagan R. Patterson S. Juergens C. Greenberg D. Givon-Lavi N. Porat N.. (2013). Comparative Immunogenicity and Efficacy of 13-Valent and 7-Valent Pneumococcal Conjugate Vaccines in Reducing Nasopharyngeal Colonization: A Randomized Double-Blind Trial. Clin. Infect. Dis. 57, 952–962. doi: 10.1093/cid/cit428 23804191

[B32] Dalia A. B. Standish A. J. Weiser J. N. (2010). Three Surface Exoglycosidases From Streptococcus Pneumoniae, NanA, BgaA, and StrH, Promote Resistance to Opsonophagocytic Killing by Human Neutrophils. Infect. Immun. 78, 2108–2116. doi: 10.1128/IAI.01125-09 20160017 PMC2863504

[B33] Daniels C. C. Coan P. King J. Hale J. Benton K. A. Briles D. E.. (2010). The Proline-Rich Region of Pneumococcal Surface Proteins A and C Contains Surface-Accessible Epitopes Common to All Pneumococci and Elicits Antibody-Mediated Protection Against Sepsis. Infect. Immun. 78, 2163–2172. doi: 10.1128/IAI.01199-09 20194601 PMC2863538

[B34] Daniels C. C. Rogers P. D. Shelton C. M. (2016). A Review of Pneumococcal Vaccines: Current Polysaccharide Vaccine Recommendations and Future Protein Antigens. J. Pediatr. Pharmacol. Ther. 21, 27–35. doi: 10.5863/1551-6776-21.1.27 26997927 PMC4778694

[B35] Dave S. Brooks-Walter A. Pangburn M. K. McDaniel L. S. (2001). PspC, a Pneumococcal Surface Protein, Binds Human Factor H. Infect. Immun. 69, 3435–3437. doi: 10.1128/IAI.69.5.3435-3437.2001 11292770 PMC98306

[B36] Denny F. W. Loda F. A. (1986). Acute Respiratory Infections are the Leading Cause of Death in Children in Developing Countries. Am. J. Trop. Med. Hyg. 35, 1–2. doi: 10.4269/ajtmh.1986.35.1 3946732

[B37] Denoël P. Godfroid F. Hermand P. Verlant V. Poolman J. (2011a). Combined Protective Effects of Anti-PhtD and Anti-Pneumococcal Polysaccharides. Vaccine 29, 6451–6453. doi: 10.1016/j.vaccine.2011.01.085 21315695

[B38] Denoël P. Philipp M. T. Doyle L. Martin D. Carletti G. Poolman J. T. (2011b). A Protein-Based Pneumococcal Vaccine Protects Rhesus Macaques From Pneumonia After Experimental Infection With Streptococcus Pneumoniae. Vaccine 29, 5495–5501. doi: 10.1016/j.vaccine.2011.05.051 21624422 PMC5061031

[B39] Dieudonné-Vatran A. Krentz S. Blom A. M. Meri S. Henriques-Normark B. Riesbeck K.. (2009). Clinical Isolates of Streptococcus Pneumoniae Bind the Complement Inhibitor C4b-Binding Protein in a PspC Allele-Dependent Fashion. J. Immunol. 182, 7865–7877. doi: 10.4049/jimmunol.0802376 19494311

[B40] Dintilhac A. Alloing G. Granadel C. Claverys J. P. (1997). Competence and Virulence of Streptococcus Pneumoniae: Adc and PsaA Mutants Exhibit a Requirement for Zn and Mn Resulting From Inactivation of Putative ABC Metal Permeases. Mol. Microbiol. 25, 727–739. doi: 10.1046/j.1365-2958.1997.5111879.x 9379902

[B41] Doyle C. R. Pirofski L. (2016). Reduction of Streptococcus Pneumoniae Colonization and Dissemination by a Nonopsonic Capsular Polysaccharide Antibody. MBio 7, e02260-15. doi: 10.1128/mBio.02260-15 26838726 PMC4742719

[B42] Doyle C. R Moon J- Y. Daily J. P Wang T. Pirofski L-a (2021). A Capsular Polysaccharide-Specific Antibody Alters Streptococcus Pneumoniae Gene Expression During Nasopharyngeal Colonization of Mice. Infect. Immun. 86, e00300-18. doi: 10.1128/IAI.00300-18 PMC6013671 29735523

[B43] Eijkelkamp B. A. Pederick V. G. Plumptre C. D. Harvey R. M. Hughes C. E. Paton J. C.. (2016). The First Histidine Triad Motif of PhtD Is Critical for Zinc Homeostasis in Streptococcus Pneumoniae. Infect. Immun. 84, 407–415. doi: 10.1128/IAI.01082-15 26573735 PMC4730578

[B44] Ekström N. Väkeväinen M. Verho J. Kilpi T. Käyhty H. (2007). Functional Antibodies Elicited by Two Heptavalent Pneumococcal Conjugate Vaccines in The Finnish Otitis Media Vaccine Trial. Infect. Immun. 75, 1794–1800. doi: 10.1128/IAI.01673-06 17261612 PMC1865725

[B45] Fabrizio K. Groner A. Boes M. Pirofski L. (2007). A Human Monoclonal Immunoglobulin M Reduces Bacteremia and Inflammation in a Mouse Model of Systemic Pneumococcal Infection. Clin. Vaccine Immunol. 14, 382–390. doi: 10.1128/CVI.00374-06 17301214 PMC1865609

[B46] Feldman C. Munro N. C. Jeffery P. K. Mitchell T. J. Andrew P. W. Boulnois G. J.. (1991). Pneumolysin Induces the Salient Histologic Features of Pneumococcal Infection in the Rat Lung In Vivo. Am. J. Respir. Cell Mol. Biol. 5, 416–423. doi: 10.1165/ajrcmb/5.5.416 1834101

[B47] Ferreira D. M. Arêas A. P. M. Darrieux M. Leite L. C. C. Miyaji E. N. (2006a). DNA Vaccines Based on Genetically Detoxified Derivatives of Pneumolysin Fail to Protect Mice Against Challenge With Streptococcus Pneumoniae. FEMS Immunol. Med. Microbiol. 46, 291–297. doi: 10.1111/j.1574-695X.2006.00040.x 16487311

[B48] Ferreira D. M. Darrieux M. Silva D. A. Leite L. C. C. Ferreira J. M. C. J. Ho P. L.. (2009). Characterization of Protective Mucosal and Systemic Immune Responses Elicited by Pneumococcal Surface Protein PspA and PspC Nasal Vaccines Against a Respiratory Pneumococcal Challenge in Mice. Clin. Vaccine Immunol. 16, 636–645. doi: 10.1128/CVI.00395-08 19279169 PMC2681601

[B49] Ferreira D. M. Miyaji E. N. Oliveira M. L. S. Darrieux M. Arêas A. P. M. Ho P. L.. (2006b). DNA Vaccines Expressing Pneumococcal Surface Protein A (PspA) Elicit Protection Levels Comparable to Recombinant Protein. J. Med. Microbiol. 55, 375–378. doi: 10.1099/jmm.0.46217-0 16533983

[B50] Fischer R. T. Longo D. L. Kenny J. J. (1995). A Novel Phosphocholine Antigen Protects Both Normal and X-Linked Immune Deficient Mice Against Streptococcus Pneumoniae. Comparison of the 6-O-Phosphocholine Hydroxyhexanoate-Conjugate With Other Phosphocholine-Containing Vaccines. J. Immunol. 154, 3373–3382. 7897220

[B51] Francis J. P. Richmond P. C. Pomat W. S. Michael A. Keno H. Phuanukoonnon S.. (2009). Maternal Antibodies to Pneumolysin But Not to Pneumococcal Surface Protein A Delay Early Pneumococcal Carriage in High-Risk Papua New Guinean Infants. Clin. Vaccine Immunol. 16, 1633–1638. doi: 10.1128/CVI.00247-09 19776196 PMC2772384

[B52] Frey S. E. Lottenbach K. R. Hill H. Blevins T. P. Yu Y. Zhang Y.. (2013). A Phase I, Dose-Escalation Trial in Adults of Three Recombinant Attenuated Salmonella Typhi Vaccine Vectors Producing Streptococcus Pneumoniae Surface Protein Antigen PspA. Vaccine 31, 4874–4880. doi: 10.1016/j.vaccine.2013.07.049 23916987

[B53] Galán-Bartual S. Pérez-Dorado I. García P. Hermoso J. A. (2015). “Chapter 11 - Structure and Function of Choline-Binding Proteins,” In: Streptococcus Pneumoniae Molecular Mechanisms of Host-Pathogen Interactions. Eds. Brown J. Hammerschmidt S. Orihuela C. B. T.-S. P. (Amsterdam: Academic Press), 207–230. doi: 10.1016/B978-0-12-410530-0.00011-9

[B54] Ganaie F. Saad J. S. McGee L. van Tonder A. J. Bentley S. D. Lo S. W.. (2020). A New Pneumococcal Capsule Type, 10D, is the 100th Serotype and Has a Large Cps Fragment From an Oral Streptococcus. MBio 11. doi: 10.1128/mBio.00937-20 PMC7240158 32430472

[B55] García-Suárez M. del M. Cima-Cabal M. D. Flórez N. García P. Cernuda-Cernuda R.. (2004). Protection Against Pneumococcal Pneumonia in Mice by Monoclonal Antibodies to Pneumolysin. Infect. Immun. 72, 4534–4540. doi: 10.1128/IAI.72.8.4534-4540.2004 15271913 PMC470670

[B56] García-Suárez M. D. M. Flórez N. Astudillo A. Vázquez F. Villaverde R. Fabrizio K.. (2007). The Role of Pneumolysin in Mediating Lung Damage in a Lethal Pneumococcal Pneumonia Murine Model. Respir. Res. 8, 3. doi: 10.1186/1465-9921-8-3 17257395 PMC1790890

[B57] Genschmer K. R. Vadesilho C. F. M. McDaniel L. S. Park S.-S. Hale Y. Miyaji E. N.. (2019). The Modified Surface Killing Assay Distinguishes Between Protective and Nonprotective Antibodies to PspA. mSphere 4, e00589–19. doi: 10.1128/mSphere.00589-19 PMC6908419 31826968

[B58] Georgieva M. Kagedan L. Lu Y.-J. Thompson C. M. Lipsitch M. (2018). Antigenic Variation in Streptococcus Pneumoniae PspC Promotes Immune Escape in the Presence of Variant-Specific Immunity. MBio 9, e00264–18. doi: 10.1128/mBio.00264-18 PMC5850329 29535198

[B59] Ginsburg A. S. Nahm M. H. Khambaty F. M. Alderson M. R. (2012). Issues and Challenges in the Development of Pneumococcal Protein Vaccines. Expert Rev. Vaccines 11, 279–285. doi: 10.1586/erv.12.5 22380821 PMC3777823

[B60] Glennie S. Gritzfeld J. F. Pennington S. H. Garner-Jones M. Coombes N. Hopkins M. J.. (2016). Modulation of Nasopharyngeal Innate Defenses by Viral Coinfection Predisposes Individuals to Experimental Pneumococcal Carriage. Mucosal Immunol. 9, 56–67. doi: 10.1038/mi.2015.35 25921341 PMC4703943

[B61] Glover D. T. Hollingshead S. K. Briles D. E. (2008). Streptococcus Pneumoniae Surface Protein PcpA Elicits Protection Against Lung Infection and Fatal Sepsis. Infect. Immun. 76, 2767–2776. doi: 10.1128/IAI.01126-07 18391008 PMC2423059

[B62] Godfroid F. Hermand P. Verlant V. Denoël P. Poolman J. T. (2011). Preclinical Evaluation of the Pht Proteins as Potential Cross-Protective Pneumococcal Vaccine Antigens. Infect. Immun. 79, 238–245. doi: 10.1128/IAI.00378-10 20956575 PMC3019885

[B63] Goldblatt D. Southern J. Ashton L. Andrews N. Woodgate S. Burbidge P.. (2010). Immunogenicity of a Reduced Schedule of Pneumococcal Conjugate Vaccine in Healthy Infants and Correlates of Protection for Serotype 6B in the United Kingdom. Pediatr. Infect. Dis. J. 29, 401–405. doi: 10.1097/INF.0b013e3181c67f04 20010312

[B64] Goulart C. da Silva T. R. Rodriguez D. Politano W. R. Leite L. C. C. Darrieux M. (2013). Characterization of Protective Immune Responses Induced by Pneumococcal Surface Protein A in Fusion With Pneumolysin Derivatives. PLoS One 8, e59605. doi: 10.1371/journal.pone.0059605 23533636 PMC3606166

[B65] Haleem K. S. Ali Y. M. Yesilkaya H. Kohler T. Hammerschmidt S. Andrew P. W.. (2019). The Pneumococcal Surface Proteins PspA and PspC Sequester Host C4-Binding Protein To Inactivate Complement C4b on the Bacterial Surface. Infect. Immun. 87, e00742–18. doi: 10.1128/IAI.00742-18 PMC6300637 30323030

[B66] Hammerschmidt S. Agarwal V. Kunert A. Haelbich S. Skerka C. Zipfel P. F. (2007). The Host Immune Regulator Factor H Interacts *via* Two Contact Sites With the PspC Protein of Streptococcus Pneumoniae and Mediates Adhesion to Host Epithelial Cells. J. Immunol. 178, 5848–5858. doi: 10.4049/jimmunol.178.9.5848 17442969

[B67] Hammitt L. L. Campbell J. C. Borys D. Weatherholtz R. C. Reid R. Goklish N.. (2019). Efficacy, Safety and Immunogenicity of a Pneumococcal Protein-Based Vaccine Co-Administered With 13-Valent Pneumococcal Conjugate Vaccine Against Acute Otitis Media in Young Children: A Phase IIb Randomized Study. Vaccine 37, 7482–7492. doi: 10.1016/j.vaccine.2019.09.076 31629570

[B68] Hammond A. J. Binsker U. Aggarwal S. D. Ortigoza M. B. Loomis C. Weiser J. N. (2021). Neuraminidase B Controls Neuraminidase A-Dependent Mucus Production and Evasion. PLoS Pathog. 17, e1009158. doi: 10.1371/journal.ppat.1009158 33819312 PMC8049478

[B69] Han C. Zhang M. (2019). Genetic Diversity and Antigenicity Analysis of Streptococcus Pneumoniae Pneumolysin Isolated From Children With Pneumococcal Infection. Int. J. Infect. Dis. 86, 57–64. doi: 10.1016/j.ijid.2019.06.025 31255709

[B70] Hermand P. Vandercammen A. Mertens E. Di Paolo E. Verlant V. Denoël P.. (2017). Preclinical Evaluation of a Chemically Detoxified Pneumolysin as Pneumococcal Vaccine Antigen. Hum. Vaccin. Immunother. 13, 220–228. doi: 10.1080/21645515.2016.1234553 27768518 PMC5287308

[B71] Hernani M. de L. Ferreira P. C. D. Ferreira D. M. Miyaji E. N. Ho P. L.. (2011). And Nasal Immunization of Mice With Lactobacillus Casei Expressing the Pneumococcal Surface Protein C Primes the Immune System and Decreases Pneumococcal Nasopharyngeal Colonization in Mice. FEMS Immunol. Med. Microbiol. 62, 263–272. doi: 10.1111/j.1574-695X.2011.00809.x 21492260

[B72] Hollingshead S. K. Becker R. Briles D. E. (2000). Diversity of PspA: Mosaic Genes and Evidence for Past Recombination in Streptococcus Pneumoniae. Infect. Immun. 68, 5889–5900. doi: 10.1128/IAI.68.10.5889-5900.2000 10992499 PMC101551

[B73] Holmlund E. Quiambao B. Ollgren J. Nohynek H. Käyhty H. (2006). Development of Natural Antibodies to Pneumococcal Surface Protein A, Pneumococcal Surface Adhesin A and Pneumolysin in Filipino Pregnant Women and Their Infants in Relation to Pneumococcal Carriage. Vaccine 24, 57–65. doi: 10.1016/j.vaccine.2005.07.055 16115703

[B74] Huang J. Gingerich A. D. Royer F. Paschall A. V. Pena-Briseno A. Avci F. Y.. (2021). Broadly Reactive Human Monoclonal Antibodies Targeting the Pneumococcal Histidine Triad Protein Protect Against Fatal Pneumococcal Infection. Infect. Immun. 89, e00747–20. doi: 10.1128/IAI.00747-20 PMC8091081 33649050

[B75] Huo Z. Spencer O. Miles J. Johnson J. Holliman R. Sheldon J.. (2004). Antibody Response to Pneumolysin and to Pneumococcal Capsular Polysaccharide in Healthy Individuals and Streptococcus Pneumoniae Infected Patients. Vaccine 22, 1157–1161. doi: 10.1016/j.vaccine.2003.09.025 15003643

[B76] Hu Y. Park N. Seo K. S. Park J. Y. Somarathne R. P. Olivier A. K.. (2021). Pneumococcal Surface Adhesion A Protein (PsaA) Interacts With Human Annexin A2 on Airway Epithelial Cells. Virulence 12, 1841–1854. doi: 10.1080/21505594.2021.1947176 34233589 PMC8274441

[B77] Hyams C. Camberlein E. Cohen J. M. Bax K. Brown J. S. (2010). The Streptococcus Pneumoniae Capsule Inhibits Complement Activity and Neutrophil Phagocytosis by Multiple Mechanisms. Infect. Immun. 78, 704–715. doi: 10.1128/IAI.00881-09 19948837 PMC2812187

[B78] Iannelli F. Oggioni M. R. Pozzi G. (2002). Allelic Variation in the Highly Polymorphic Locus pspC of Streptococcus Pneumoniae. Gene 284, 63–71. doi: 10.1016/s0378-1119(01)00896-4 11891047

[B79] Iuchi H. Ohori J. Kyutoku T. Ito K. Kurono Y. (2019). Role of Phosphorylcholine in Streptococcus Pneumoniae and Nontypeable Haemophilus Influenzae Adherence to Epithelial Cells. Auris. Nasus. Larynx. 46, 513–519. doi: 10.1016/j.anl.2018.11.003 30503566

[B80] Jahn K. Handtke S. Palankar R. Weißmüller S. Nouailles G. Kohler T. P.. (2020). Pneumolysin Induces Platelet Destruction, Not Platelet Activation, Which can be Prevented by Immunoglobulin Preparations In Vitro. Blood Adv. 4, 6315–6326. doi: 10.1182/bloodadvances.2020002372 33351126 PMC7756997

[B81] Jakubovics N. S. Jenkinson H. F. (2001). Out of the Iron Age: New Insights Into the Critical Role of Manganese Homeostasis in Bacteria. Microbiology 147, 1709–1718. doi: 10.1099/00221287-147-7-1709 11429449

[B82] Janesch P. Rouha H. Badarau A. Stulik L. Mirkina I. Caccamo M.. (2018). Assessing the Function of Pneumococcal Neuraminidases NanA, NanB and NanC in In Vitro and *In Vivo* Lung Infection Models Using Monoclonal Antibodies. Virulence 9, 1521–1538. doi: 10.1080/21505594.2018.1520545 30289054 PMC6177239

[B83] Johnston J. W. Briles D. E. Myers L. E. Hollingshead S. K. (2006). Mn2+-Dependent Regulation of Multiple Genes in Streptococcus Pneumoniae Through PsaR and the Resultant Impact on Virulence. Infect. Immun. 74, 1171–1180. doi: 10.1128/IAI.74.2.1171-1180.2006 16428766 PMC1360317

[B84] Kallio A. Sepponen K. Hermand P. Denoël P. Godfroid F. Melin M. (2014). Role of Pht Proteins in Attachment of Streptococcus Pneumoniae to Respiratory Epithelial Cells. Infect. Immun. 82, 1683–1691. doi: 10.1128/IAI.00699-13 24491577 PMC3993382

[B85] Kamtchoua T. Bologa M. Hopfer R. Neveu D. Hu B. Sheng X.. (2013). Safety and Immunogenicity of the Pneumococcal Pneumolysin Derivative PlyD1 in a Single-Antigen Protein Vaccine Candidate in Adults. Vaccine 31, 327–333. doi: 10.1016/j.vaccine.2012.11.005 23153437

[B86] Kanclerski K. Möllby R. (1987). Production and Purification of Streptococcus Pneumoniae Hemolysin (Pneumolysin). J. Clin. Microbiol. 25, 222–225. doi: 10.1128/jcm.25.2.222-225.1987 3818918 PMC265871

[B87] Kaur R. Surendran N. Ochs M. Pichichero M. E. (2014). Human Antibodies to PhtD, PcpA, and Ply Reduce Adherence to Human Lung Epithelial Cells and Murine Nasopharyngeal Colonization by Streptococcus Pneumoniae. Infect. Immun. 82, 5069–5075. doi: 10.1128/IAI.02124-14 25245804 PMC4249272

[B88] Kazemian H. Afshar D. Garcia E. Pourmand M. R. Jeddi-Tehrani M. Aminharati F.. (2018). CbpM and CbpG of Streptococcus Pneumoniae Elicit a High Protection in Mice Challenged With a Serotype 19f Pneumococcus. Iran. J. Allergy Asthma. Immunol. 17, 574–585. 30644702

[B89] Keller L. E. Robinson D. A. McDaniel L. S. (2016). Nonencapsulated Streptococcus Pneumoniae: Emergence and Pathogenesis. MBio 7, e01792. doi: 10.1128/mBio.01792-15 27006456 PMC4807366

[B90] Kerr A. R. Paterson G. K. McCluskey J. Iannelli F. Oggioni M. R. Pozzi G.. (2006). The Contribution of PspC to Pneumococcal Virulence Varies Between Strains and is Accomplished by Both Complement Evasion and Complement-Independent Mechanisms. Infect. Immun. 74, 5319–5324. doi: 10.1128/IAI.00543-06 16926426 PMC1594871

[B91] Khan N. Jan A. T. (2017). Towards Identifying Protective B-Cell Epitopes: The PspA Story. Front. Microbiol. 8. doi: 10.3389/fmicb.2017.00742 PMC5411445 28512452

[B92] Khan M. N. Pichichero M. E. (2012). Vaccine Candidates PhtD and PhtE of Streptococcus Pneumoniae are Adhesins That Elicit Functional Antibodies in Humans. Vaccine 30, 2900–2907. doi: 10.1016/j.vaccine.2012.02.023 22349524 PMC3490617

[B93] Khan N. Qadri R. A. Sehgal D. (2015). Correlation Between In Vitro Complement Deposition and Passive Mouse Protection of Anti-Pneumococcal Surface Protein A Monoclonal Antibodies. Clin. Vaccine Immunol. 22, 99–107. doi: 10.1128/CVI.00001-14 25410204 PMC4278925

[B94] Khan M. N. Sharma S. K. Filkins L. M. Pichichero M. E. (2012). PcpA of Streptococcus Pneumoniae Mediates Adherence to Nasopharyngeal and Lung Epithelial Cells and Elicits Functional Antibodies in Humans. Microbes Infect. 14, 1102–1110. doi: 10.1016/j.micinf.2012.06.007 22796387 PMC3490615

[B95] Kharat A. S. Tomasz A. (2006). Drastic Reduction in the Virulence of Streptococcus Pneumoniae Expressing Type 2 Capsular Polysaccharide But Lacking Choline Residues in the Cell Wall. Mol. Microbiol. 60, 93–107. doi: 10.1111/j.1365-2958.2006.05082.x 16556223

[B96] King S. J. Hippe K. R. Weiser J. N. (2006). Deglycosylation of Human Glycoconjugates by the Sequential Activities of Exoglycosidases Expressed by Streptococcus Pneumoniae. Mol. Microbiol. 59, 961–974. doi: 10.1111/j.1365-2958.2005.04984.x 16420364

[B97] Klugman K. P. (2009). The Significance of Serotype Replacement for Pneumococcal Disease and Antibiotic Resistance. Adv. Exp. Med. Biol. 634, 121–128. doi: 10.1007/978-0-387-79838-7_11 19280854

[B98] Kohler S. Hallström T. Singh B. Riesbeck K. Spartà G. Zipfel P. F.. (2015). Binding of Vitronectin and Factor H to Hic Contributes to Immune Evasion of Streptococcus Pneumoniae Serotype 3. Thromb. Haemost. 113, 125–142. doi: 10.1160/TH14-06-0561 25181963

[B99] Kristian S. A. Ota T. Bubeck S. S. Cho R. Groff B. C. Kubota T.. (2016). Generation and Improvement of Effector Function of a Novel Broadly Reactive and Protective Monoclonal Antibody Against Pneumococcal Surface Protein A of Streptococcus Pneumoniae. PLoS One 11, e0154616. doi: 10.1371/journal.pone.0154616 27171010 PMC4865217

[B100] Kucinskaite-Kodze I. Simanavicius M. Dapkunas J. Pleckaityte M. Zvirbliene A. (2020). Mapping of Recognition Sites of Monoclonal Antibodies Responsible for the Inhibition of Pneumolysin Functional Activity. Biomol 10. doi: 10.3390/biom10071009 PMC7408604 32650398

[B101] Kumar S. Sunagar R. Gosselin E. J. (2020). Preclinical Efficacy of a Trivalent Human Fcγri-Targeted Adjuvant-Free Subunit Mucosal Vaccine Against Pulmonary Pneumococcal Infection. Vaccines 8, 193. doi: 10.3390/vaccines8020193 PMC7349865 32340134

[B102] Lawrence M. C. Pilling P. A. Epa V. C. Berry A. M. Ogunniyi A. D. Paton J. C. (1998). The Crystal Structure of Pneumococcal Surface Antigen PsaA Reveals a Metal-Binding Site and a Novel Structure for a Putative ABC-Type Binding Protein. Structure 6, 1553–1561. doi: 10.1016/s0969-2126(98)00153-1 9862808

[B103] Lee H. Choi E. H. Lee H. J. (2014). Efficacy and Effectiveness of Extended-Valency Pneumococcal Conjugate Vaccines. Korean. J. Pediatr. 57, 55–66. doi: 10.3345/kjp.2014.57.2.55 24678328 PMC3965795

[B104] Leroux-Roels G. Maes C. De Boever F. Traskine M. Rüggeberg J. U. Borys D. (2014). Safety, Reactogenicity and Immunogenicity of a Novel Pneumococcal Protein-Based Vaccine in Adults: A Phase I/II Randomized Clinical Study. Vaccine 32, 6838–6846. doi: 10.1016/j.vaccine.2014.02.052 24607003

[B105] Linley E. Bell A. Gritzfeld J. F. Borrow R. (2019). Should Pneumococcal Serotype 3 Be Included in Serotype-Specific Immunoassays? Vaccines 7, 4. doi: 10.3390/vaccines7010004 PMC6466091 30609868

[B106] Li Y. Weinberger D. M. Thompson C. M. Trzciński K. Lipsitch M. (2013). Surface Charge of Streptococcus Pneumoniae Predicts Serotype Distribution. Infect. Immun. 81, 4519–4524. doi: 10.1128/IAI.00724-13 24082068 PMC3837974

[B107] Luck J. N. Tettelin H. Orihuela C. J. (2020). Sugar-Coated Killer: Serotype 3 Pneumococcal Disease. Front. Cell. Infect. Microbiol. 10. doi: 10.3389/fcimb.2020.613287 PMC7786310 33425786

[B108] Lu L. Ma Z. Jokiranta T. S. Whitney A. R. DeLeo F. R. Zhang J.-R. (2008). Species-Specific Interaction of Streptococcus Pneumoniae With Human Complement Factor H. J. Immunol. 181, 7138–7146. doi: 10.4049/jimmunol.181.10.7138 18981135 PMC2587499

[B109] Lu J. Sun T. Hou H. Xu M. Gu T. Dong Y.. (2014). Detoxified Pneumolysin Derivative Plym2 Directly Protects Against Pneumococcal Infection via Induction of Inflammatory Cytokines. Immunol. Invest. 43, 717–726. doi: 10.3109/08820139.2014.930478 25020076

[B110] Magee A. D. Yother J. (2001). Requirement for Capsule in Colonization by Streptococcus Pneumoniae. Infect. Immun. 69, 3755–3761. doi: 10.1128/IAI.69.6.3755-3761.2001 11349040 PMC98386

[B111] Malekan M. Siadat S. D. Aghasadeghi M. Shahrokhi N. Afrough P. Behrouzi A.. (2020). Evaluation of Protective Immunity Responses Against Pneumococcal PhtD and its C-Terminal in Combination With Outer-Membrane Vesicles as Adjuvants. J. Med. Microbiol. 69, 465–477. doi: 10.1099/jmm.0.001103 32100705

[B112] Mann B. Orihuela C. Antikainen J. Gao G. Sublett J. Korhonen T. K.. (2006). Multifunctional Role of Choline Binding Protein G in Pneumococcal Pathogenesis. Infect. Immun. 74, 821–829. doi: 10.1128/IAI.74.2.821-829.2006 16428724 PMC1360319

[B113] Mann B. Thornton J. Heath R. Wade K. R. Tweten R. K. Gao G.. (2014). Broadly Protective Protein-Based Pneumococcal Vaccine Composed of Pneumolysin Toxoid-CbpA Peptide Recombinant Fusion Protein. J. Infect. Dis. 209, 1116–1125. doi: 10.1093/infdis/jit502 24041791 PMC3952665

[B114] Marks L. R. Reddinger R. M. Hakansson A. P. (2014). Biofilm Formation Enhances Fomite Survival of Streptococcus Pneumoniae and Streptococcus Pyogenes. Infect. Immun. 82, 1141–1146. doi: 10.1128/IAI.01310-13 24371220 PMC3957990

[B115] Martinez P. J. Farhan A. Mustafa M. Javaid N. Darkoh C. Garrido-Sanabria E.. (2019). PspA Facilitates Evasion of Pneumococci From Bactericidal Activity of Neutrophil Extracellular Traps (NETs). Microb. Pathog. 136, 103653. doi: 10.1016/j.micpath.2019.103653 31398527

[B116] McAllister L. J. Tseng H. Ogunniyi A. D. Jennings M. P. McEwan A. G. Paton J. C. (2004). And Molecular Analysis of the Psa Permease Complex of Streptococcus Pneumoniae. Mol. Microbiol. 53, 889–901. doi: 10.1111/j.1365-2958.2004.04164.x 15255900

[B117] McCool T. L. Cate T. R. Moy G. Weiser J. N. (2002). The Immune Response to Pneumococcal Proteins During Experimental Human Carriage. J. Exp. Med. 195, 359–365. doi: 10.1084/jem.20011576 11828011 PMC2193593

[B118] Mirza S. Benjamin W. H. J. Coan P. A. Hwang S.-A. Winslett A.-K. Yother J.. (2016). The Effects of Differences in pspA Alleles and Capsular Types on the Resistance of Streptococcus Pneumoniae to Killing by Apolactoferrin. Microb. Pathog. 99, 209–219. doi: 10.1016/j.micpath.2016.08.029 27569531

[B119] Mitchell T. J. Andrew P. W. Saunders F. K. Smith A. N. Boulnois G. J. (1991). Complement Activation and Antibody Binding by Pneumolysin *via* a Region of the Toxin Homologous to a Human Acute-Phase Protein. Mol. Microbiol. 5, 1883–1888. doi: 10.1111/j.1365-2958.1991.tb00812.x 1766369

[B120] Mitsi E. Roche A. M. Reiné J. Zangari T. Owugha J. T. Pennington S. H.. (2017). Agglutination by Anti-Capsular Polysaccharide Antibody is Associated With Protection Against Experimental Human Pneumococcal Carriage. Mucosal Immunol. 10, 385–394. doi: 10.1038/mi.2016.71 27579859 PMC5332540

[B121] Miyaji E. N. Dias W. O. Gamberini M. Gebara V. C. B. C. Schenkman R. P. F. Wild J.. (2001). PsaA (Pneumococcal Surface Adhesin A) and PspA (Pneumococcal Surface Protein A) DNA Vaccines Induce Humoral and Cellular Immune Responses Against Streptococcus Pneumoniae. Vaccine 20, 805–812. doi: 10.1016/S0264-410X(01)00395-4 11738744

[B122] Moffitt K. Malley R. (2016). Rationale and Prospects for Novel Pneumococcal Vaccines. Hum. Vaccin. Immunother. 12, 383–392. doi: 10.1080/21645515.2015.1087625 26535755 PMC5049723

[B123] Mold C. Du Clos T. W. Nakayama S. Edwards K. M. Gewurz H. (1982). C-Reactive Protein Reactivity With Complement and Effects on Phagocytosis. Ann. N. Y. Acad. Sci. 389, 251–262. doi: 10.1111/j.1749-6632.1982.tb22141.x 7046579

[B124] Mukerji R. Mirza S. Roche A. M. Widener R. W. Croney C. M. Rhee D.-K.. (2012). Pneumococcal Surface Protein A Inhibits Complement Deposition on the Pneumococcal Surface by Competing With the Binding of C-Reactive Protein to Cell-Surface Phosphocholine. J. Immunol. 189, 5327–5335. doi: 10.4049/jimmunol.1201967 23105137 PMC3517878

[B125] Nabors G. S. Braun P. A. Herrmann D. J. Heise M. L. Pyle D. J. Gravenstein S.. (2000). Immunization of Healthy Adults With a Single Recombinant Pneumococcal Surface Protein A (PspA) Variant Stimulates Broadly Cross-Reactive Antibodies to Heterologous PspA Molecules. Vaccine 18, 1743–1754. doi: 10.1016/s0264-410x(99)00530-7 10699322

[B126] Nagano H. Kawabata M. Sugita G. Tsuruhara A. Ohori J. Jimura T.. (2018). Transcutaneous Immunization With Pneumococcal Surface Protein A in Mice. Laryngoscope 128, E91–E96. doi: 10.1002/lary.26971 29226330

[B127] Nakahashi-Ouchida R. Uchida Y. Yuki Y. Katakai Y. Yamanoue T. Ogawa H.. (2021). A Nanogel-Based Trivalent PspA Nasal Vaccine Protects Macaques From Intratracheal Challenge With Pneumococci. Vaccine 39, 3353–3364. doi: 10.1016/j.vaccine.2021.04.069 34016473

[B128] Nelson A. L. Roche A. M. Gould J. M. Chim K. Ratner A. J. Weiser J. N. (2007). Capsule Enhances Pneumococcal Colonization by Limiting Mucus-Mediated Clearance. Infect. Immun. 75, 83–90. doi: 10.1128/IAI.01475-06 17088346 PMC1828419

[B129] Novak R. Tuomanen E. Charpentier E. (2000). The Mystery of psaA and Penicillin Tolerance in Streptococcus Pneumoniae: MicroCorrespondence. Mol. Microbiol. 36, 1505–1506. doi: 10.1046/j.1365-2958.2000.01959.x 10947276

[B130] O’Brien K. L. Wolfson L. J. Watt J. P. Henkle E. Deloria-Knoll M. McCall N.. (2009). Burden of Disease Caused by Streptococcus Pneumoniae in Children Younger Than 5 Years: Global Estimates. Lancet (London. England). 374, 893–902. doi: 10.1016/S0140-6736(09)61204-6 19748398

[B131] Ochs M. M. Williams K. Sheung A. Lheritier P. Visan L. Rouleau N.. (2016). A Bivalent Pneumococcal Histidine Triad Protein D-Choline-Binding Protein A Vaccine Elicits Functional Antibodies That Passively Protect Mice From Streptococcus Pneumoniae Challenge. Hum. Vaccin. Immunother. 12, 2946–2952. doi: 10.1080/21645515.2016.1202389 27392182 PMC5137517

[B132] Odutola A. Ota M. O. C. Antonio M. Ogundare E. O. Saidu Y. Foster-Nyarko E.. (2017). Efficacy of a Novel, Protein-Based Pneumococcal Vaccine Against Nasopharyngeal Carriage of Streptococcus Pneumoniae in Infants: A Phase 2, Randomized, Controlled, Observer-Blind Study. Vaccine 35, 2531–2542. doi: 10.1016/j.vaccine.2017.03.071 28389097

[B133] Ogunniyi A. D. Grabowicz M. Mahdi L. K. Cook J. Gordon D. L. Sadlon T. A.. (2009). Pneumococcal Histidine Triad Proteins are Regulated by the Zn2+-Dependent Repressor AdcR and Inhibit Complement Deposition Through the Recruitment of Complement Factor H. FASEB J. 23, 731–738. doi: 10.1096/fj.08-119537 18971260

[B134] Oliveira M. L. S. Arêas A. P. M. Campos I. B. Monedero V. Perez-Martínez G. Miyaji E. N.. (2006). Induction of Systemic and Mucosal Immune Response and Decrease in Streptococcus Pneumoniae Colonization by Nasal Inoculation of Mice With Recombinant Lactic Acid Bacteria Expressing Pneumococcal Surface Antigen A. Microbes Infect. 8, 1016–1024. doi: 10.1016/j.micinf.2005.10.020 16549380 PMC7110601

[B135] Orihuela C. J. Mahdavi J. Thornton J. Mann B. Wooldridge K. G. Abouseada N.. (2009). Laminin Receptor Initiates Bacterial Contact With the Blood Brain Barrier in Experimental Meningitis Models. J. Clin. Invest. 119, 1638–1646. doi: 10.1172/JCI36759 19436113 PMC2689107

[B136] Papastamatiou T. Routsias J. G. Koutsoni O. Dotsika E. Tsakris A. Spoulou V. (2018). Evaluation of Protective Efficacy of Selected Immunodominant B-Cell Epitopes Within Virulent Surface Proteins of Streptococcus Pneumoniae. Infect. Immun. 86, e00673–17. doi: 10.1128/IAI.00673-17 PMC5820952 29263108

[B137] Parker D. Soong G. Planet P. Brower J. Ratner A. J. Prince A. (2009). The NanA Neuraminidase of Streptococcus Pneumoniae is Involved in Biofilm Formation. Infect. Immun. 77, 3722–3730. doi: 10.1128/IAI.00228-09 19564377 PMC2738052

[B138] Paton J. C. Rowan-Kelly B. Ferrante A. (1984). Activation of Human Complement by the Pneumococcal Toxin Pneumolysin. Infect. Immun. 43, 1085–1087. doi: 10.1128/iai.43.3.1085-1087.1984 6698602 PMC264298

[B139] Petukhova E. S. Vorobyev D. S. Sidorov A. V. Semenova I. B. Volokh Y. V. Leonova A. Y.. (2020). Immunization With Recombinant Pneumolysin Induces the Production of Antibodies and Protects Mice in a Model of Systemic Infection Caused by Streptococcus Pneumoniae. Bull. Exp. Biol. Med. 168, 485–487. doi: 10.1007/s10517-020-04736-6 32146631

[B140] Piao Z. Akeda Y. Takeuchi D. Ishii K. J. Ubukata K. Briles D. E.. (2014). Protective Properties of a Fusion Pneumococcal Surface Protein A (PspA) Vaccine Against Pneumococcal Challenge by Five Different PspA Clades in Mice. Vaccine 32, 5607–5613. doi: 10.1016/j.vaccine.2014.07.108 25132335

[B141] Pimenta F. C. Miyaji E. N. Arêas A. P. M. Oliveira M. L. S. De Andrade A. Ho P. L.. (2006). Intranasal Immunization With the Cholera Toxin B Subunit-Pneumococcal Surface Antigen A Fusion Protein Induces Protection Against Colonization With Streptococcus Pneumoniae and has Negligible Impact on the Nasopharyngeal and Oral Microbiota of Mice. Infect. Immun. 74, 4939–4944. doi: 10.1128/IAI.00134-06 16861686 PMC1539618

[B142] Plumptre C. D. Ogunniyi A. D. Paton J. C. (2013a). Surface Association of Pht Proteins of Streptococcus Pneumoniae. Infect. Immun. 81, 3644–3651. doi: 10.1128/IAI.00562-13 23876799 PMC3811752

[B143] Plumptre C. D. Ogunniyi A. D. Paton J. C. (2013b). Vaccination Against Streptococcus Pneumoniae Using Truncated Derivatives of Polyhistidine Triad Protein D. PLoS One 8, e78916. doi: 10.1371/journal.pone.0078916 24205351 PMC3814962

[B144] Poehling K. A. Talbot T. R. Griffin M. R. Craig A. S. Whitney C. G. Zell E.. (2006). Invasive Pneumococcal Disease Among Infants Before and After Introduction of Pneumococcal Conjugate Vaccine. JAMA 295, 1668–1674. doi: 10.1001/jama.295.14.1668 16609088

[B145] Prevaes S. M. P. J. van Wamel W. J. B. de Vogel C. P. Veenhoven R. H. van Gils E. J. M. van Belkum A.. (2012). Nasopharyngeal Colonization Elicits Antibody Responses to Staphylococcal and Pneumococcal Proteins That Are Not Associated With a Reduced Risk of Subsequent Carriage. Infect. Immun. 80 (6), 2186–2193. doi: 10.1128/IAI.00037-12 22451514 PMC3370583

[B146] Price K. E. Camilli A. (2009). Pneumolysin Localizes to the Cell Wall of Streptococcus Pneumoniae. J. Bacteriol. 191, 2163–2168. doi: 10.1128/JB.01489-08 19168620 PMC2655535

[B147] Prymula R. Hanovcova I. Splino M. Kriz P. Motlova J. Lebedova V.. (2011). Impact of the 10-Valent Pneumococcal non-Typeable Haemophilus Influenzae Protein D Conjugate Vaccine (PHiD-CV) on Bacterial Nasopharyngeal Carriage. Vaccine 29, 1959–1967. doi: 10.1016/j.vaccine.2010.12.086 21215830

[B148] Ravinder K. Naveen S. Martina O. E P. M. A M. B. (2014). Human Antibodies to PhtD, PcpA, and Ply Reduce Adherence to Human Lung Epithelial Cells and Murine Nasopharyngeal Colonization by Streptococcus Pneumoniae. Infect. Immun. 82, 5069–5075. doi: 10.1128/IAI.02124-14 25245804 PMC4249272

[B149] Rayner C. F. Jackson A. D. Rutman A. Dewar A. Mitchell T. J. Andrew P. W.. (1995). Interaction of Pneumolysin-Sufficient and -Deficient Isogenic Variants of Streptococcus Pneumoniae With Human Respiratory Mucosa. Infect. Immun. 63, 442–447. doi: 10.1128/iai.63.2.442-447.1995 7822008 PMC173015

[B150] Ren B. Li J. Genschmer K. Hollingshead S. K. Briles D. E. (2012). The Absence of PspA or Presence of Antibody to PspA Facilitates the Complement-Dependent Phagocytosis of Pneumococci *In Vitro* . Clin. Vaccine Immunol. 19, 1574–1582. doi: 10.1128/CVI.00393-12 22855389 PMC3485889

[B151] Ren B. Szalai A. J. Hollingshead S. K. Briles D. E. (2004). Effects of PspA and Antibodies to PspA on Activation and Deposition of Complement on The Pneumococcal Surface. Infect. Immun. 72, 114–122. doi: 10.1128/IAI.72.1.114-122.2004 14688088 PMC344006

[B152] Ricci S. Janulczyk R. Gerlini A. Braione V. Colomba L. Iannelli F.. (2011). The Factor H-Binding Fragment of PspC as a Vaccine Antigen for the Induction of Protective Humoral Immunity Against Experimental Pneumococcal Sepsis. Vaccine 29, 8241–8249. doi: 10.1016/j.vaccine.2011.08.119 21911026

[B153] Ring A. Weiser J. N. Tuomanen E. I. (1998). Pneumococcal Trafficking Across the Blood-Brain Barrier. Molecular Analysis of a Novel Bidirectional Pathway. J. Clin. Invest. 102, 347–360. doi: 10.1172/JCI2406 9664076 PMC508893

[B154] Romero-Steiner S. Caba J. Rajam G. Langley T. Floyd A. Johnson S. E.. (2006). Adherence of Recombinant Pneumococcal Surface Adhesin A (Rpsaa)-Coated Particles to Human Nasopharyngeal Epithelial Cells for the Evaluation of Anti-PsaA Functional Antibodies. Vaccine 24, 3224–3231. doi: 10.1016/j.vaccine.2006.01.042 16487631

[B155] Romero-Steiner S. Libutti D. Pais L. B. Dykes J. Anderson P. Whitin J. C.. (1997). Standardization of an Opsonophagocytic Assay for the Measurement of Functional Antibody Activity Against Streptococcus Pneumoniae Using Differentiated HL-60 Cells. Clin. Diagn. Lab. Immunol. 4, 415–422. doi: 10.1128/cdli.4.4.415-422.1997 9220157 PMC170543

[B156] Romero-Steiner S. Pilishvili T. Sampson J. S. Johnson S. E. Stinson A. Carlone G. M.. (2003). Inhibition of Pneumococcal Adherence to Human Nasopharyngeal Epithelial Cells by Anti-PsaA Antibodies. Clin. Diagn. Lab. Immunol. 10, 246–251. doi: 10.1128/cdli.10.2.246-251.2003 12626450 PMC150525

[B157] Rosenow C. Ryan P. Weiser J. N. Johnson S. Fontan P. Ortqvist A.. (1997). Contribution of Novel Choline-Binding Proteins to Adherence, Colonization and Immunogenicity of Streptococcus Pneumoniae. Mol. Microbiol. 25, 819–829. doi: 10.1111/j.1365-2958.1997.mmi494.x 9364908

[B158] Rubins J. B. Charboneau D. Paton J. C. Mitchell T. J. Andrew P. W. Janoff E. N. (1995). Dual Function of Pneumolysin in the Early Pathogenesis of Murine Pneumococcal Pneumonia. J. Clin. Invest. 95, 142–150. doi: 10.1172/JCI117631 7814608 PMC295392

[B159] Rubins J. B. Duane P. G. Charboneau D. Janoff E. N. (1992). Toxicity of Pneumolysin to Pulmonary Endothelial Cells *In Vitro* . Infect. Immun. 60, 1740–1746. doi: 10.1128/iai.60.5.1740-1746.1992 1563759 PMC257067

[B160] Sampson J. S. Furlow Z. Whitney A. M. Williams D. Facklam R. Carlone G. M. (1997). Limited Diversity of Streptococcus Pneumoniae psaA Among Pneumococcal Vaccine Serotypes. Infect. Immun. 65, 1967–1971. doi: 10.1128/iai.65.5.1967-1971.1997 9125591 PMC175255

[B161] Sampson J. S. O’Connor S. P. Stinson A. R. Tharpe J. A. Russell H. (1994). Cloning and Nucleotide Sequence Analysis of Psaa, the Streptococcus Pneumoniae Gene Encoding a 37-Kilodalton Protein Homologous to Previously Reported Streptococcus Sp. Adhesins. Infect. Immun. 62, 319–324. doi: 10.1128/iai.62.1.319-324.1994 7505262 PMC186105

[B162] Sánchez-Beato A. R. López R. García J. L. (1998). Molecular Characterization of PcpA: A Novel Choline-Binding Protein of Streptococcus Pneumoniae. FEMS Microbiol. Lett. 164, 207–214. doi: 10.1111/j.1574-6968.1998.tb13087.x 9675866

[B163] Sanders M. E. Norcross E. W. Moore Q. C. 3rd Fratkin J. Thompson H. Marquart M. E. (2010). Immunization With Pneumolysin Protects Against Both Retinal and Global Damage Caused by Streptococcus Pneumoniae Endophthalmitis. J. Ocul. Pharmacol. Ther. 26, 571–577. doi: 10.1089/jop.2010.0077 21034245 PMC2990286

[B164] Sarah W. Haijun T. Nico van R. Liise-anne P. N W. J. (2012). A Serotype 3 Pneumococcal Capsular Polysaccharide-Specific Monoclonal Antibody Requires Fcγ Receptor III and Macrophages To Mediate Protection Against Pneumococcal Pneumonia in Mice. Infect. Immun. 80, 1314–1322. doi: 10.1128/IAI.06081-11 22290146 PMC3318404

[B165] Seiberling M. Bologa M. Brookes R. Ochs M. Go K. Neveu D.. (2012). Safety and Immunogenicity of a Pneumococcal Histidine Triad Protein D Vaccine Candidate in Adults. Vaccine 30, 7455–7460. doi: 10.1016/j.vaccine.2012.10.080 23131206

[B166] Shak J. R. Ludewick H. P. Howery K. E. Sakai F. Yi H. Harvey R. M.. (2013). Novel Role for the Streptococcus Pneumoniae Toxin Pneumolysin in the Assembly of Biofilms. MBio 4, e00655-13. doi: 10.1128/mBio.00655-13 24023386 PMC3774193

[B167] Shaper M. Hollingshead S. K. Benjamin W. H. J. Briles D. E. (2004). PspA Protects Streptococcus Pneumoniae From Killing by Apolactoferrin, and Antibody To PspA Enhances Killing of Pneumococci by Apolactoferrin [Corrected]. Infect. Immun. 72, 5031–5040. doi: 10.1128/IAI.72.9.5031-5040.2004 15321996 PMC517438

[B168] Simell B. Auranen K. Käyhty H. Goldblatt D. Dagan R. O’Brien K. L. (2012). The Fundamental Link Between Pneumococcal Carriage and Disease. Expert Rev. Vaccines 11, 841–855. doi: 10.1586/erv.12.53 22913260

[B169] Song J. Y. Moseley M. A. Burton R. L. Nahm M. H. (2013). Pneumococcal Vaccine and Opsonic Pneumococcal Antibody. J. Infect. Chemother. 19, 412–425. doi: 10.1007/s10156-013-0601-1 23657429 PMC3692352

[B170] Steinfort C. Wilson R. Mitchell T. Feldman C. Rutman A. Todd H.. (1989). Effect of Streptococcus Pneumoniae on Human Respiratory Epithelium. Vitro. Infect. Immun. 57, 2006–2013. doi: 10.1128/iai.57.7.2006-2013.1989 2731981 PMC313834

[B171] Subramanian K. Neill D. R. Malak H. A. Spelmink L. Khandaker S. Dalla Libera Marchiori G.. (2019). Pneumolysin Binds to the Mannose Receptor C Type 1 (MRC-1) Leading to Anti-Inflammatory Responses and Enhanced Pneumococcal Survival. Nat. Microbiol. 4, 62–70. doi: 10.1038/s41564-018-0280-x 30420782 PMC6298590

[B172] Sundberg-Kövamees M. Holme T. Sjögren A. (1996). Interaction of the C-Polysaccharide of Streptococcus Pneumoniae With the Receptor Asialo-GM1. Microb. Pathog. 21, 223–234. doi: 10.1006/mpat.1996.0057 8905612

[B173] Swiatlo E. King J. Nabors G. S. Mathews B. Briles D. E. (2003). PneumococcalSurface Protein A Is Expressed In Vivo, and Antibodies to PspA AreEffective for Therapy in a Murine Model of PneumococcalSepsis. Infect. Immun. 71, 7149–7153. doi: 10.1128/IAI.71.12.7149-7153.2003 14638806 PMC308907

[B174] Talkington D. F. Brown B. G. Tharpe J. A. Koenig A. Russell H. (1996). Protection of Mice Against Fatal Pneumococcal Challenge by Immunization With Pneumococcal Surface Adhesin A (PsaA). Microb. Pathog. 21, 17–22. doi: 10.1006/mpat.1996.0038 8827703

[B175] Tanaka N. Fukuyama S. Fukuiwa T. Kawabata M. Sagara Y. Ito H.. (2007). Intranasal Immunization With Phosphorylcholine Induces Antigen Specific Mucosal and Systemic Immune Responses in Mice. Vaccine 25, 2680–2687. doi: 10.1016/j.vaccine.2006.10.014 17270319

[B176] Thanawastien A. Joyce K. E. Cartee R. T. Haines L. A. Pelton S. I. Tweten R. K.. (2021). Preclinical In Vitro and *In Vivo* Profile of a Highly-Attenuated, Broadly Efficacious Pneumolysin Genetic Toxoid. Vaccine 39, 1652–1660. doi: 10.1016/j.vaccine.2020.04.064 32532546 PMC8237519

[B177] Tian H. Weber S. Thorkildson P. Kozel T. R. Pirofski L.-A. (2009). Efficacy of Opsonic and Nonopsonic Serotype 3 Pneumococcal Capsular Polysaccharide-Specific Monoclonal Antibodies Against Intranasal Challenge With Streptococcus Pneumoniae in Mice. Infect. Immun. 77, 1502–1513. doi: 10.1128/IAI.01075-08 19168739 PMC2663166

[B178] Toh Z. Q. Higgins R. A. Mazarakis N. Abbott E. Nathanielsz J. Balloch A.. (2021). Evaluating Functional Immunity Following Encapsulated Bacterial Infection and Vaccination. Vaccines 9, 667. doi: 10.3390/vaccines9060677 34203030 PMC8234458

[B179] Trolle S. Chachaty E. Kassis-Chikhani N. Wang C. Fattal E. Couvreur P.. (2000). Intranasal Immunization With Protein-Linked Phosphorylcholine Protects Mice Against a Lethal Intranasal Challenge With Streptococcus Pneumoniae. Vaccine 18, 2991–2998. doi: 10.1016/S0264-410X(00)00089-X 10825601

[B180] Tseng H.-J. McEwan A. G. Paton J. C. Jennings M. P. (2002). Virulence of Streptococcus Pneumoniae: PsaA Mutants are Hypersensitive to Oxidative Stress. Infect. Immun. 70, 1635–1639. doi: 10.1128/IAI.70.3.1635-1639.2002 11854257 PMC127802

[B181] Tu A. H. Fulgham R. L. McCrory M. A. Briles D. E. Szalai A. J. (1999). Pneumococcal Surface Protein A Inhibits Complement Activation by Streptococcus Pneumoniae. Infect. Immun. 67, 4720–4724. doi: 10.1128/IAI.67.9.4720-4724.1999 10456922 PMC96800

[B182] Turner P. Turner C. Green N. Ashton L. Lwe E. Jankhot A.. (2013). Serum Antibody Responses to Pneumococcal Colonization in the First 2 Years of Life: Results From an SE Asian Longitudinal Cohort Study. Clin. Microbiol. Infect. 19, E551–E558. doi: 10.1111/1469-0691.12286 24255996 PMC4282116

[B183] Uchiyama S. Carlin A. F. Khosravi A. Weiman S. Banerjee A. Quach D.. (2009). The Surface-Anchored NanA Protein Promotes Pneumococcal Brain Endothelial Cell Invasion. J. Exp. Med. 206, 1845–1852. doi: 10.1084/jem.20090386 19687228 PMC2737157

[B184] Verhoeven D. Xu Q. Pichichero M. E. (2014). Vaccination With a Streptococcus Pneumoniae Trivalent Recombinant PcpA, PhtD and PlyD1 Protein Vaccine Candidate Protects Against Lethal Pneumonia in an Infant Murine Model. Vaccine 32, 3205–3210. doi: 10.1016/j.vaccine.2014.04.004 24731814

[B185] Visan L. Rouleau N. Proust E. Peyrot L. Donadieu A. Ochs M. (2018). Antibodies to PcpA and PhtD Protect Mice Against Streptococcus Pneumoniae by a Macrophage- and Complement-Dependent Mechanism. Hum. Vaccin. Immunother. 14, 489–494. doi: 10.1080/21645515.2017.1403698 29135332 PMC5806646

[B186] von Gottberg A. de Gouveia L. Tempia S. Quan V. Meiring S. von Mollendorf C.. (2014). Effects of Vaccination on Invasive Pneumococcal Disease in South Africa. N. Engl. J. Med. 371, 1889–1899. doi: 10.1056/NEJMoa1401914 25386897

[B187] Voss S. Hallström T. Saleh M. Burchhardt G. Pribyl T. Singh B.. (2013). The Choline-Binding Protein PspC of Streptococcus Pneumoniae Interacts With the C-Terminal Heparin-Binding Domain of Vitronectin. J. Biol. Chem. 288, 15614–15627. doi: 10.1074/jbc.M112.443507 23603906 PMC3668722

[B188] Wallick S. Claflin J. L. Briles D. E. (1983). Resistance to Streptococcus Pneumoniae is Induced by a Phosphocholine-Protein Conjugate. J. Immunol. 130, 2871–2875. 6854020

[B189] Wang S. Li Y. Shi H. Scarpellini G. Torres-Escobar A. Roland K. L.. (2010). Immune Responses to Recombinant Pneumococcal PsaA Antigen Delivered by a Live Attenuated Salmonella Vaccine. Infect. Immun. 78, 3258–3271. doi: 10.1128/IAI.00176-10 20479086 PMC2897375

[B190] Wantuch P. L. Avci F. Y. (2018). Current Status and Future Directions of Invasive Pneumococcal Diseases and Prophylactic Approaches to Control Them. Hum. Vaccin. Immunother. 14, 2303–2309. doi: 10.1080/21645515.2018.1470726 29757699 PMC6183136

[B191] Wartha F. Beiter K. Albiger B. Fernebro J. Zychlinsky A. Normark S.. (2007). Capsule and D-Alanylated Lipoteichoic Acids Protect Streptococcus Pneumoniae Against Neutrophil Extracellular Traps. Cell. Microbiol. 9, 1162–1171. doi: 10.1111/j.1462-5822.2006.00857.x 17217430

[B192] Weinberger D. M. Dagan R. Givon-Lavi N. Regev-Yochay G. Malley R. Lipsitch M. (2008). Epidemiologic Evidence for Serotype-Specific Acquired Immunity to Pneumococcal Carriage. J. Infect. Dis. 197, 1511–1518. doi: 10.1086/587941 18471062

[B193] Weiser J. N. Ferreira D. M. Paton J. C. (2018). Streptococcus Pneumoniae: Transmission, Colonization and Invasion. Nat. Rev. Microbiol. 16, 355–367. doi: 10.1038/s41579-018-0001-8 29599457 PMC5949087

[B194] W J. J. E M. L. M O. M. H B. W. E B. D. K H. S. (2004). Lipoprotein PsaA in Virulence of Streptococcus Pneumoniae: Surface Accessibility and Role in Protection From Superoxide. Infect. Immun. 72, 5858–5867. doi: 10.1128/IAI.72.10.5858-5867.2004 15385487 PMC517531

[B195] WHO (2017). WHO Publishes List of Bacteria for Which New Antibiotics are Urgently Needed (Geneva: Media Cent).

[B196] Wiedinger K. McCauley J. Bitsaktsis C. (2020). Isotype-Specific Outcomes in Fc Gamma Receptor Targeting of PspA Using Fusion Proteins as a Vaccination Strategy Against Streptococcus Pneumoniae Infection. Vaccine 38, 5634–5646. doi: 10.1016/j.vaccine.2020.06.067 32646816

[B197] Wilson R. Cohen J. M. Reglinski M. Jose R. J. Chan W. Y. Marshall H.. (2017). Naturally Acquired Human Immunity to Pneumococcus Is Dependent on Antibody to Protein Antigens. PLoS Pathog. 13, e1006137. doi: 10.1371/journal.ppat.1006137 28135322 PMC5279798

[B198] Wizemann T. M. Heinrichs J. H. Adamou J. E. Erwin A. L. Kunsch C. Choi G. H.. (2001). Use of a Whole Genome Approach to Identify Vaccine Molecules Affording Protection Against Streptococcus Pneumoniae Infection. Infect. Immun. 69, 1593–1598. doi: 10.1128/IAI.69.3.1593-1598.2001 11179332 PMC98061

[B199] Wu H. Y. Nahm M. H. Guo Y. Russell M. W. Briles D. E. (1997). Intranasal Immunization of Mice With PspA (Pneumococcal Surface Protein A) can Prevent Intranasal Carriage, Pulmonary Infection, and Sepsis With Streptococcus Pneumoniae. J. Infect. Dis. 175, 839–846. doi: 10.1086/513980 9086139

[B200] Xu G. Kiefel M. J. Wilson J. C. Andrew P. W. Oggioni M. R. Taylor G. L. (2011). Three Streptococcus Pneumoniae Sialidases: Three Different Products. J. Am. Chem. Soc 133, 1718–1721. doi: 10.1021/ja110733q 21244006

[B201] Xu Q. Pryharski K. Pichichero M. E. (2017). Trivalent Pneumococcal Protein Vaccine Protects Against Experimental Acute Otitis Media Caused by Streptococcus Pneumoniae in an Infant Murine Model. Vaccine 35, 337–344. doi: 10.1016/j.vaccine.2016.11.046 27919628

[B202] Yahiaoui R. Y. den Heijer C. D. van Bijnen E. M. Paget W. J. Pringle M. Goossens H.. (2016). Prevalence and Antibiotic Resistance of Commensal Streptococcus Pneumoniae in Nine European Countries. Future Microbiol. 11, 737–744. doi: 10.2217/fmb-2015-0011 27191588

[B203] Yano M. Gohil S. Coleman J. R. Manix C. Pirofski L. (2011). Antibodies to Streptococcus Pneumoniae Capsular Polysaccharide Enhance Pneumococcal Quorum Sensing. MBio 2, e00176–11. doi: 10.1128/mBio.00176-11 PMC3171983 21917597

[B204] Yoo I.-H. Shin H.-S. Kim Y.-J. Kim H.-B. Jin S. Ha U.-H. (2010). Role of Pneumococcal Pneumolysin in the Induction of an Inflammatory Response in Human Epithelial Cells. FEMS Immunol. Med. Microbiol. 60, 28–35. doi: 10.1111/j.1574-695X.2010.00699.x 20528932

[B205] Yun K. W. Lee H. Choi E. H. Lee H. J. (2015). Diversity of Pneumolysin and Pneumococcal Histidine Triad Protein D of Streptococcus Pneumoniae Isolated From Invasive Diseases in Korean Children. PLoS One 10, e0134055. doi: 10.1371/journal.pone.0134055 26252211 PMC4529296

[B206] Zafar M. A. Wang Y. Hamaguchi S. Weiser J. N. (2017). Host-To-Host Transmission of Streptococcus Pneumoniae Is Driven by Its Inflammatory Toxin, Pneumolysin. Cell Host Microbe 21, 73–83. doi: 10.1016/j.chom.2016.12.005 28081446 PMC5267320

[B207] Zhang Q. Bernatoniene J. Bagrade L. Pollard A. J. Mitchell T. J. Paton J. C.. (2006). Serum and Mucosal Antibody Responses to Pneumococcal Protein Antigens in Children:Relationships With Carriage Status. Eur. J. Immunol. 36, 46–57. doi: 10.1002/eji.200535101 16342325

[B208] Zysk G. Schneider-Wald B. K. Hwang J. H. Bejo L. Kim K. S. Mitchell T. J.. (2001). Pneumolysin is the Main Inducer of Cytotoxicity to Brain Microvascular Endothelial Cells Caused by Streptococcus Pneumoniae. Infect. Immun. 69, 845–852. doi: 10.1128/IAI.69.2.845-852.2001 11159977 PMC97961

